# Asymmetric Interplay Between K^+^ and Blocker and Atomistic Parameters From Physiological Experiments Quantify K^+^ Channel Blocker Release

**DOI:** 10.3389/fphys.2021.737834

**Published:** 2021-10-29

**Authors:** Tobias S. Gabriel, Ulf-Peter Hansen, Martin Urban, Nils Drexler, Tobias Winterstein, Oliver Rauh, Gerhard Thiel, Stefan M. Kast, Indra Schroeder

**Affiliations:** ^1^Plant Membrane Biophysics, Technische Universität Darmstadt, Darmstadt, Germany; ^2^Department of Structural Biology, Christian-Albrechts-Universität zu Kiel, Kiel, Germany; ^3^Physikalische Chemie III, Technische Universita̋t Dortmund, Dortmund, Germany; ^4^Institute of Physiology II, University Hospital Jena, Friedrich Schiller University Jena, Jena, Germany

**Keywords:** selectivity filter, ion binding, blocker kinetics, 3D RISM, extended beta distributions, viral potassium channels, carbonyl-ion interaction

## Abstract

Modulating the activity of ion channels by blockers yields information on both the mode of drug action and on the biophysics of ion transport. Here we investigate the interplay between ions in the selectivity filter (SF) of K^+^ channels and the release kinetics of the blocker tetrapropylammonium in the model channel Kcv_NTS_. A quantitative expression calculates blocker release rate constants directly from voltage-dependent ion occupation probabilities in the SF. The latter are obtained by a kinetic model of single-channel currents recorded in the absence of the blocker. The resulting model contains only two adjustable parameters of ion-blocker interaction and holds for both symmetric and asymmetric ionic conditions. This data-derived model is corroborated by 3D reference interaction site model (3D RISM) calculations on several model systems, which show that the K^+^ occupation probability is unaffected by the blocker, a direct consequence of the strength of the ion-carbonyl attraction in the SF, independent of the specific protein background. Hence, Kcv_NTS_ channel blocker release kinetics can be reduced to a small number of system-specific parameters. The pore-independent asymmetric interplay between K^+^ and blocker ions potentially allows for generalizing these results to similar potassium channels.

## Introduction

Ion channels are crucial for many cellular functions and therefore important drug targets (Ashcroft, 2006; Bernard and Shevell, 2008; Fernández-Ballester et al., 2011; Bagal et al., 2013). A major class of drugs for potassium (K^+^) channels are pore blockers that bind in the aqueous cavity between selectivity filter and intracellular pore entrance. Many of these molecules are either positively charged (Sánchez-Chapula et al., 2002) or bind with a positively charge moiety oriented towards the selectivity filter (Bucchi et al., 2013; Du et al., 2014). Examples are the heart rate-reducing agent ivabradine for HCN channels (Bucchi et al., 2013), and drugs blocking hERG channels either as a desired effect (e.g., the antiarrhythmic ranolazine, Du et al., 2014) or as an unwanted side effect, like Chloroquine (Sánchez-Chapula et al., 2002). Due to the structural conservation within the superfamily of cation-selective channels, similar findings have been reported for other channels, e.g., a block of Na_V_ channels by different anticonvulsants and anesthetics (Tikhonov and Zhorov, 2017). To facilitate the development of new compounds, a profound understanding of the molecular interactions between the blocker, the channel protein, and the permeating ions is essential.

Decades of research with pore blockers (Armstrong and Hille, 1972; Villarroel et al., 1988; Choi et al., 1993; Jara-Oseguera et al., 2007; Piechotta et al., 2011) have contributed to a detailed understanding of the channels’ structure and dynamics including the insight that mutual interactions between blocker and permeating ions are crucial for blocker binding/release kinetics. Two examples are the release of a positively charged blocker from TRPV (Jara-Oseguera et al., 2007) and MthK (Posson et al., 2013) channels, which is in both cases accelerated via the electrostatic repulsion by nearby cations.

However, a quantitative model for blocker release kinetics in a K^+^ channel that explicitly considers the distinct population states in the selectivity filter (SF) is still missing. To this end, it is necessary to measure the voltage-dependent rate constants of blocker release, and to determine the occupation probabilities of the ion binding sites in the SF. So far, these ion occupation probabilities can only be provided by computational modeling via MD simulations (Roux, 2005; Köpfer et al., 2014), liquid state theory (Kast et al., 2011), or experimentally by X-ray crystallography (Morais-Cabral et al., 2001; Yohannan et al., 2007), though only under non-physiological conditions and in most cases without the crucial voltage dependency. Considering the uncertainties of the underlying model assumptions (Roux, 2005; Köpfer et al., 2014; Rauh et al., 2018), ion distributions should ideally be determined experimentally under the same physiological conditions employed in the blocking experiments.

Here we apply methods developed previously (Rauh et al., 2018), which allow for determining the required voltage-dependent ion occupation probabilities of the individual binding sites in the SF of a K^+^ channel from experimental open-state data. This was achieved by a global fit of single-channel current-voltage relationships and gating kinetics in the SF of a model K^+^ channel (Kcv_NTS_, Rauh et al., 2018) using an appropriate flux model (Roux, 2005) from the MD simulation literature. From the rate constants of ion hopping in this model, the desired voltage-dependent ion occupancies of the binding sites in the SF can be calculated.

Combined with a kinetic model of blocker release that depends on these calculated local SF occupancies obtained from *open-state* kinetic constants we end up with a quantitative expression for the voltage-dependent kinetic release constant. This model requires only two adjustable parameters that can be taken from experimental data on the voltage dependency of blocker release under symmetric ionic conditions, as exemplified for Kcv_NTS_ blocked by tetrapropylammonium (TPrA). The robustness of the expression is corroborated in two ways. First, the model is shown to be also applicable quantitatively to recordings under asymmetric bath conditions. Second, we validate the assumption that the presence of the blocker has no effect on SF populations and, therefore, does not influence the free energy surface governing the kinetic constants of K^+^ transport. Such an insensitivity of the SF populations to the presence of blockers has already been found in several studies of crystal structures (Faraldo-Gómez et al., 2007; Yohannan et al., 2007; Lenaeus et al., 2014). Here, we provide further evidence from calculations using the three-dimensional reference interaction site model (3D RISM) integral equation theory. This approach provides ion and solvent populations for a given SF structure including permeating ions and the charged blocker. The results obtained for a variety of filter structures, including a maximally reduced system comprising only the canonical SF of K^+^ channels, show conclusively that neither the blocker nor the protein environment affect SF occupancies. This can be traced back to the peculiar nature of K^+^ SF energetics. Hence, the combined experimental and computational data uncover an asymmetric interplay between the blocker and K^+^ ions in the filter which allows for transferring independently gathered open-state kinetic data to a blocking kinetics model, a key result of the present work. In this context, the term “asymmetry” does of course not mean a violation of force balance between interacting partners, but compactly describes the apparent strong effect of filter ion confinement to decouple occupancies from the blocker ion presence, facilitating the construction of a kinetic model in which the blocker simply switches off certain ion transitions in the flux model.

The K^+^ channel Kcv_NTS_ used for the experimental studies represents the pore module of all K^+^ channels, including the canonical SF sequence. Hence, we expect that the robust observation of ion/blocker asymmetry from the maximally reduced computational model translates to other K^+^ channels. Together with the abstraction of blocker release kinetics to only two system-specific parameters, this implies that the phenomenon of asymmetric filter ion/blocker interplay may also hold for mammalian K^+^ channels where this insight may ultimately improve the possibilities of rational drug design.

## Materials and Methods

### *In vitro* Protein Expression and Purification

The Kcv_NTS_ protein was expressed *in vitro* and purified as described previously (Rauh et al., 2017b). Briefly, the gene of Kcv_NTS_ (Greiner, 2011; Jeanniard et al., 2013) was cloned into a pEXP5-CT/TOPO^®^-vector (Invitrogen, Karlsbad, CA, United States), the fusion of the His tag coded for in the plasmid was prevented by inserting a stop codon.

*In vitro* expression of the channel protein was performed with the MembraneMax^TM^ *HN* Protein Expression Kit (Invitrogen) following the manufacturer’s instructions. During the expression procedure, the Kcv_NTS_ proteins were directly embedded into nanolipoproteins (NLPs), containing multiple His-tags (Katzen et al., 2008). This allows the purification of the native Kcv_NTS_ protein by metal chelate affinity chromatography. Purification was done on a 0.2 mL HisPur^TM^ Ni-NTA spin column (Thermo Fisher Scientific, Waltham, MA, United States). To improve the reconstitution efficiency into the bilayer (Winterstein et al., 2018), neither the washing nor the elution solutions contained salts. The column was washed three times with two resin-bed volumes of 20 mM imidazole to remove unspecific binders. The Kcv_NTS_-containing NLPs were eluted in three fractions (200 μL each) with 250 mM imidazole.

### Lipid Bilayer Experiments

Planar lipid bilayer experiments at room temperature (20-25°C) were performed on a vertical bilayer setup (IonoVation, Osnabrück, Germany) as described previously (Braun et al., 2013). Briefly, 1,2-diphytanoyl-*sn*-glycero-3-phosphocholine (DPhPC, Avanti Polar Lipids, Alabaster, AL, United States) bilayers were formed using the pseudo painting/air bubble technique (Braun et al., 2014). One of the elution fractions was diluted in 250 mM imidazole solution by a factor of 1000 to 100000. For reconstitution of the channel, a small amount (1-3 μL) of the diluted NLP/Kcv_NTS_-conjugates was added directly below the bilayer in the *trans* compartment with a bent Hamilton syringe.

Both the *cis* and *trans* compartment were filled with 100 mM KCl plus 10 mM HEPES adjusted to pH 7.0 with KOH. The correct insertion of the protein and its orientation was tested by short voltage pulses utilizing asymmetry of the apparent IV curves of Kcv channels (Gazzarrini et al., 2009). The *trans* compartment was grounded and membrane voltages were applied to the *cis* compartment. Positive currents in the graphs correspond to outward currents in the *in vivo* situation.

Constant voltages between +160 mV and −160 mV in steps of 20 mV were applied for 1 or 2 min. The single-channel current was measured via Ag/AgCl electrodes connected to the headstage of a patch-clamp amplifier (L/M-EPC-7, List-Medical, Darmstadt and Axopatch 200B, Molecular Devices). In order to prevent electromagnetic interference from outside sources, the amplifier and the 16-bit A/D converter were connected via an optical link. Currents were filtered with a 1-kHz 4-pole Bessel filter and digitized with a sampling frequency of 5 kHz (LIH 1600, HEKA Elektronik, Lambrecht, Germany). TPrA concentration was changed by replacing an appropriate amount of the solution in the *cis* (=cytosolic) chamber by a 1, 10, or 100 mM TPrA stock solution and thorough mixing. All blocker stock solutions contained 100 mM KCl plus 10 mM HEPES, pH was adjusted to 7.0 with KOH.

For the experiments with 500 mM external KCl, an appropriate amount of a KCl stock solution (3M KCl, 10 mM HEPES, pH 7.0) was added to the *trans* (=external) recording chamber after the reconstitution of a single channel. In these experiments, agar bridges (3M or 500 mM KCl, 1.5% agarose) were used for both electrodes.

### Determination of *I*_true_ and the Rate Constants of Fast Gating From Extended Beta Distribution Analysis

If the rate constants of blocking or flickering are higher than the bandwidth of the low-pass filter of the experimental set-up, the related changes in current are strongly attenuated. In other words, neither the individual gating transitions, nor the true open channel current, *I*_*true*_, can be directly observed. However, the attenuated flickering still causes “excess noise” (Heinemann and Sigworth, 1991; Schroeder, 2015), which results in broadened, non-Gaussian peaks in the amplitude histograms (Supplementary Figure S1). From these curves, the hidden gating parameters can be extracted by extended beta distribution analysis (Schroeder, 2015; Rauh et al., 2017a, 2018). The simulation algorithm for the generation of artificial time series of current and the equations for the calculation of amplitude histograms from these time series are given in Albertsen and Hansen (1994), Schroeder and Hansen (2006). The theoretical histograms were fitted to the measured ones by a simplex algorithm (Caceci and Cacheris, 1984).

The fitting strategies have also been described elsewhere (Schroeder and Hansen, 2009; Schroeder, 2015). Here, it is important to note, that simulation is done in continuous time and includes the same baseline noise as the experiment and a digital representation of the jump-response of the 4th-order Bessel filter used for the experiments. Details of the application of this analysis to fast gating in Kcv_NTS_ have been described previously (Rauh et al., 2017a).

The fit and calculation of confidence intervals in Figure 3D was done with Mathematica.

### Computational Details

#### Structure Preparation

The protein structure of the KcsA-Fab-TBA complex (pdb code: 2HVK, Yohannan et al., 2007) was directly obtained from the protein data bank (PDB). For 3D RISM calculations only the chain consisting of the KcsA channel (chain C) and TBA (residue TBA) were used. Based on the geometric data provided in the pdb file a complete tetrameric structure was generated. All ions and water molecules were deleted, whereas for studying the effect of K^+^ at position S4 on blocker energetics the respective ion was retained at the crystallographic position. Missing protein protons were added by VMD PSFGEN plugin (Humphrey et al., 1996). All titratable residues remained in the standard ionization state. Missing TBA protons were added by “reduce” (Case et al., 2016). For the reduced template model, only the filter carbonyls (residues 74 to 79) and the TBA molecule from 2HVK were retained. All other reduced models (KcsA 1K4C, Zhou et al., 2001); KirBac3.1 3ZRS, (Bavro et al., 2012); and Kcv_PBCV–1_, (Tayefeh et al., 2009; Hoffgaard et al., 2015) were prepared similarly by aligning the filter structures to 2HVK by VMD (Humphrey et al., 1996) and keeping the TBA atom positions at the 2HVK reference values.

#### Reference Interaction Site Model Calculations

All RISM calculations were performed using software developed in ourlaboratory. For the RISM calculations models for 1 M KCl and purewater were used, applying the TIP3P water model (Jorgensen et al., 1983) with modified Lennard-Jones parameters for water of *σ* = 0.4 Å, ε = 0.0459 kcal mol^–1^ and ion parameters taken from CHARMM (Beglov and Roux, 1994). To derive the individual solvent susceptibilities 1D RISM calculations on a logarithmic grid ranging from 5.98⋅10^–3^ Å to 164.02 Å with a total of 512 points were carried out. The solvent density of 0.0333295 Å^–3^ for pure water and 0.0323666 Å^–3^ for 1 M KCl at 298.15 K were used. For both solvents a dielectric permittivity of 78.4 was applied. All 3D RISM calculations were performed on a 100 Å × 100 Å × 144 Å grid with a spacing of 0.4 Å using the 3^rd^ order Partial Series Expansion (PSE-3) closure (Kast and Kloss, 2008). 3D RISM equations were solved applying a maximum residual norm of direct correlation functions between successive steps of 10^–4^ as a convergence criterion. Solute-solvent interactions were modeled by the sum of Lennard-Jones (LJ, using standard Lorentz-Bertelot mixing rules) and Coulomb interactions. CHARMM27 (MacKerell et al., 1998) LJ parameters and partial charges for the protein were used. The partial charges of TBA were calculated using antechamber (Case et al., 2016) with AM1-BCC charges. TBA LJ parameters were obtained from general amber force field GAFF (Wang et al., 2004), see Supplementary Table S2. The calculations with reduced filter charges were performed by halving the carbonyl atom charges while keeping the total charge of the protein constant. This leads to a partial charge of the carbonyl C atom from original 0.51 to 0.255 and the carbonyl O atom from −0.51 to −0.255 e (Supplementary Table S3). Radial integration was performed based on grid data sliced by applying the HOLE algorithm (Smart et al., 1996). All parameters and structures of reduced filter models are collected in Supplementary Tables S2-S7, structures and parameters are also available in machine-readable format.

For computing the excess chemical potential change upon blocker release of 2HVK with and without an explicitly placed K^+^ at S4, we subjected four systems: (i) isolated channel, (ii) channel with K^+^, (iii) channel with TBA, and (iv) channel with TBA plus K^+^ to 3D RISM calculations in modified SPC/E water. For these calculations a quantitatively optimized solvation free energy model based on the partial molar volume (PMV) and net charge correction is available from our earlier work (Tielker et al., 2018, 2021). Following the strategy described there for pure force field-based 3D RISM calculations the parameters for scaling the PMV and the net charge term were *c*_*V*_ = −0.09982 kcal mol^–1^ Å^–3^ and *c*_*q*_ = −15.66344 kcal mol^–1^ e^–1^.

## Results and Discussion

### Electrophysiological Measurements of Blocking by Tetrapropylammonium

**FIGURE 1:**
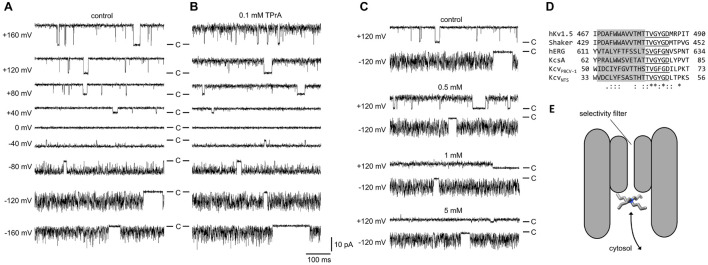
Block of single-channel currents in Kcv_NTS_ in DPhPC membranes by cytosolic TPrA. **(A,B)** Representative time series measured in symmetrical 100 mM KCl at different membrane voltages between +160 mV and −160 mV **(A)** without TPrA and **(B)** with 0.1 mM TPrA. **(C)** Time series measured in different concentrations of TPrA at +120 and −120 mV. The scale bar refers to all three panels in this figure. C marks the closed state. The data for ±120 mV without TPrA are displayed in both **(A,C)**. **(D)** Alignment of the pore loop of two viral K^+^ channels (Kcv_NTS_, Rauh et al., 2017b), the one used in this study, and Kcv_PBCV–1_ (Tayefeh et al., 2009), a bacterial K^+^ channel (KcsA, UniProtKB/Swiss-Prot: P0A334.1) and three eukaryotic potassium channels (*Shaker*, GenBank: CAA29917.1, hERG, GenBank: BAA37096.1, and human Kv1.5, NCBI Reference Sequence: NP_002225.2). Sequence conservation (^∗^identity, : conservative,. semi-conservative) was calculated with the Clustal Omega Webapp. The selectivity filter sequence is underlined, the pore helix is shaded in gray. **(E)** Schematic illustration of the blocking process, the binding site is only accessible from the cytosolic pore entrance.

In previous studies we have used the analysis of excess noise in the model channel Kcv_NTS_ for understanding the mechanism of fast gating in the SF and for determining the voltage-dependent occupation of the K^+^ binding sites in the SF (Rauh et al., 2018). Here, we employ the same analysis for a better understanding of mutual interactions between the pore blocker TPrA and ions in the SF. It is well established that quaternary ammonium ions occlude the selectivity filter from the cytosolic side (Faraldo-Gómez et al., 2007; Lenaeus et al., 2014; Figure 1E). Because of the strong conservation of the canonical selectivity filter sequence throughout potassium channels in all realms of life (Heginbotham et al., 1994; Figure 1D), it is expected that the observed effects ought to be fairly similar throughout the whole K^+^ channel family.

Under control conditions (Figure 1A), the time series of current in the Kcv_NTS_ channel display the typical high open probability with some short closures at positive voltages. At negative voltages, the current becomes increasingly noisy (“flickering,” with excess noise, Heinemann and Sigworth, 1991) due to a fast gating process in the SF (Rauh et al., 2017a, 2018). Averaging over these events makes the apparent (average) current *I*_*app*_ smaller than the actual open-channel current *I*_*true*_ (Schroeder, 2015).

At moderate TPrA concentrations (0.1 mM, Figure 1B), *I*_app_ is slightly decreased, and some flickering and increased open-channel noise occurs at positive voltages indicating fast blocking events that are not directly resolved. Higher TPrA concentrations (Figure 1C) further decrease the apparent current at positive voltages. At negative voltages, the effect is much smaller. This is expected, since the positively charged blocker has to diffuse against the inward flow of K^+^ ions, and it is influenced by interaction with an increasing ion population in the SF close to the blocker as shown below.

### Evaluation of the Rate Constants of Fast Blocking by Fitting Amplitude Histograms

**FIGURE 2:**
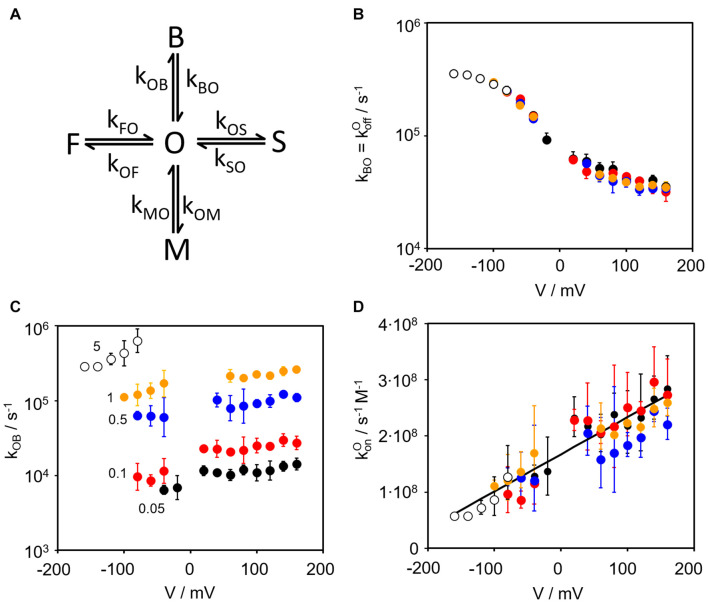
Kinetics of the TPrA block. **(A)** Kinetic Markov model used for the fits of the amplitude histograms (Supplementary Figure S1). The states O (open) and F, M, S (three closed states of different dwell times) are those used previously in the 4-state model of gating in the unblocked Kcv_NTS_ (Rauh et al., 2017a, 2018). The blocked state B is introduced by the blocker TPrA. **(B)** Voltage dependence of the rate constant $k_{\text{BO}}=k_{\text{off}}^{\text{O}}$ measured at cytosolic TPrA concentrations of 0.05 mM ●, 0.1 mM 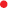, 0.5 mM 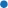, 1 mM 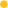, and 5 mM ο. **(C)** Voltage dependence of $k_{\text{OB}}=k_{\text{on}}^{\text{O}}\left[\text{TPrA}\right]$ measured at the same concentrations as *k*_BO_, **(D)** Voltage dependence of $k_{\text{on}}^{\text{O}}$ as determined by dividing *k*_OB_ by the concentration of TPrA in mol. Data points are the geometric mean of 3 to 4 individual channels, error bars represent the geometric standard deviation. Some data points around 0 mV are missing because of insufficient signal-to-noise ratio.

The TPrA-induced increase in open-channel noise can be exploited to extract the rate constants of blocking. The amplitude histograms (Supplementary Figure S1) are fitted with an adequate Markov model (Figure 2A). The model for gating of Kcv_NTS_ in the absence of a blocker (Schroeder, 2015; Rauh et al., 2017a, 2018) is extended by a fourth non-conducting state (B = blocked) to account for TPrA-induced block; the related rate constants are named *k*_OB_ and *k*_BO_. *k*_BO_ is equivalent to $k_{\text{off}}^{\text{O}}$ as it describes blocker dissociation in the open channel. This simplified model, in which the blocker interacts only with the open state, is justified since blocking events in single-channel recordings are only obtained for the open channel, in line with the 3D RISM calculations below that only consider structural information for the interaction of the blocker with the open channel. Any blocking events, which may occur while one of the intrinsic gates (S, M, F) is closed, do not contribute to the recorded single-channel currents and hence do not add to the amplitude histograms (Supplementary Figure S1). For the same reason they also do not occur in the model of Figure 2A. Any additional state-dependent rate constants of blocking would only be relevant if the blocker interferes with the intrinsic gates. A detailed discussion of this possibility is presented in the Supplementary Information (Supplementary Figure S4), which concludes that the influence of these secondary effects on the determination of the average rate constant $k_{\text{off}}^{\text{O}}$ is in this case so small that it can be ignored. Consequently, we use the simplified Markov model in Figure 2A for determining the rate constant $k_{\text{off}}^{\text{O}}=k_{\text{BO}}$ of blocker release in the open channel. As we here deal with the rate constant of blocker release, details of blocker binding *k*_OB_ (Figures 2C,D) are discussed in the Supplementary Material (Equations S2, S3).

Figure 2B shows that $k_{\text{off}}^{\text{O}}$ does not depend on TPrA concentration. This is not surprising because the dissociation of the blocker from its binding site in the channel cavity should not depend on the blocker concentration in the cytosol. Thus, the complete curve of the voltage dependency of $k_{\text{off}}^{\text{O}}$ can be composed from the contributions of the different TPrA concentrations. The strong overlap of data points at voltages more positive than -80 mV justifies this procedure (Figure 2B). The data underpin the choice of TPrA for these experiments as its voltage-dependent rate constants of binding and unbinding (between 7000 s^–1^ and 700000 s^–1^) are in a temporal range that is completely covered by our fast gating analysis (Schroeder, 2015). This is not the case for other QA blockers we tested.

### Correlating Voltage Dependence of the Rate Constant of Blocker Dissociation With Ion Occupation Probability in S4

**FIGURE 3:**
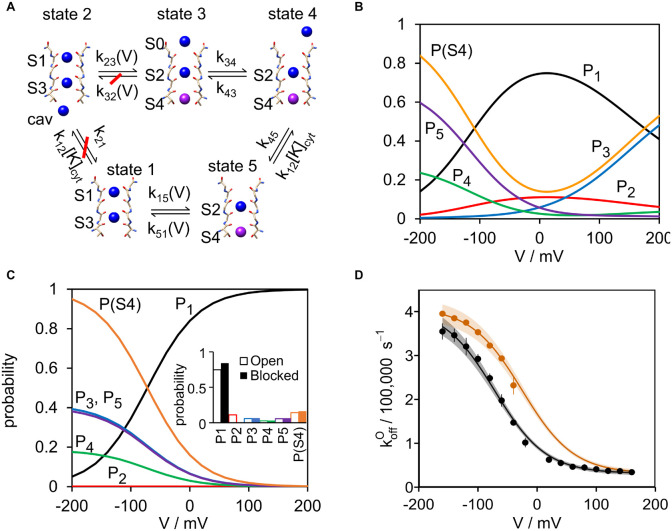
Relating the voltage dependence of the rate constant of blocker release in the open state ($k_{\text{off}}^{\text{O}}$) to the ion distribution at S4 adjacent to the binding site of the blocker. **(A)** 5-state model of ion hopping (Roux, 2005) with the rate constants *k*_*ij*_ (*i*, *j* = 1 to 5) obtained from the previous analysis (Rauh et al., 2018). Numerical values for the rate constants are given in Supplementary Table S1 **(B,C)**. The probabilities *P*_*m*_ (*m* = 1 to 5) of the occurrence of the states 1 to 5 in the model of Roux (2005) calculated from Eqs. S4 to S9 for the case **(B)** that the channel is open and not blocked (redrawn from the original data of Rauh et al., 2018) and **(C)** that the channel is blocked (*k*_12_ = *k*_21_ = *k*_32_ = 0). *P*(S4) is the probability that there is an ion in binding site S4 (purple in panel **A**) close to the binding site of the blocker. According to panel **(A)**, *P*(S4) = *P*_3_ + *P*_4_ + *P*_5_. The inset shows the probabilities for the open and blocked channel at 0 mV, as taken from panels **(B,C)**. **(D)** Comparison of the measured $k_{\text{off}}^{\text{O}}$ with the values predicted by Eq. 4 based on *P*(S4). Black line: Fit of the experimentally determined $k_{\text{off}}^{\text{O}}$ (black circles, pooled from all TPrA concentrations) with 100 mM symmetric KCl. Parameters *a* and *b* were free fit parameter in Eq. 3. Orange line: Prediction of Eq. 3 for 100 mM cytosolic and 500 mM external KCl. Parameters *a* and *b* were taken from the symmetric fit. Orange circles: experimentally determined $k_{\text{off}}^{\text{O}}$ (with 5 mM TPrA). Data points show mean and standard deviation, shaded areas depict 99% confidence intervals.

Since S4 is the ion binding site nearest to the blocker it is expected that its occupation probability *P*(S4) dominates the repulsion of the blocker. Thus, determination of *P*(S4) is required for the development of a quantitative model, which describes its effect on the rate constant $k_{\text{off}}^{\text{O}}$ of blocker dissociation. In a previous study (Rauh et al., 2018), it has been shown that the “soft knock-on” model of ion transport in the selectivity filter (Figure 3A) as developed from MD simulations (Roux, 2005) is adequate for describing ion hopping in the open Kcv_NTS_ channel. In contrast, the alternative “direct knock-on” model (Köpfer et al., 2014; Mironenko et al., 2021) was unable to describe the experimental data of Kcv_NTS_ for the K^+^ concentrations and membrane voltages used in the previous (Rauh et al., 2018) and present study, and is thus not further considered here. This does not imply that the direct knock-on model does not apply to ion channels especially as crystallographic support for a hard knock-on model has been provided (Langan et al., 2018) by anomalous X-ray diffraction studies of ion transport in K^+^ channels. However, it may suggest that different models apply to different channels. The rate constants for Figure 3A have been obtained from a global fit of IV curves and the voltage dependency of *k*_*OM*_, the rate constant of channel closure of the sub-millisecond gating in the SF, measured in symmetric 100 mM K^+^ (activity 77 mM) (Rauh et al., 2018). From these rate constants, the occupation probability curves *P*_1_ to *P*_5_ (Figure 3A) are calculated for the unblocked state (Figure 3B). The respective equations and parameters are reported in the Supplementary Material.

An important premise of the calculation of *P*(S4) in the blocked state is the insensitivity of the crucial rate constants of ion hopping (Figure 3A) to the blocker. The analysis of the crystal structure of KcsA (Faraldo-Gómez et al., 2007; Yohannan et al., 2007; Lenaeus et al., 2014) shows that the blocker does indeed not influence the ion distribution in the filter. This implies that the rate constants of ion hopping in Figure 3A, the determinants of voltage-dependent ion distribution, are not affected by the blocker. These findings are further validated by 3D RISM calculations below. While both the crystal structures and 3D RISM calculations consider the situation at 0 mV, we show in the Supplementary Material that the rate constants of ion hopping are not affected by the blocker even under the action of non-zero membrane voltage.

**FIGURE 4:**
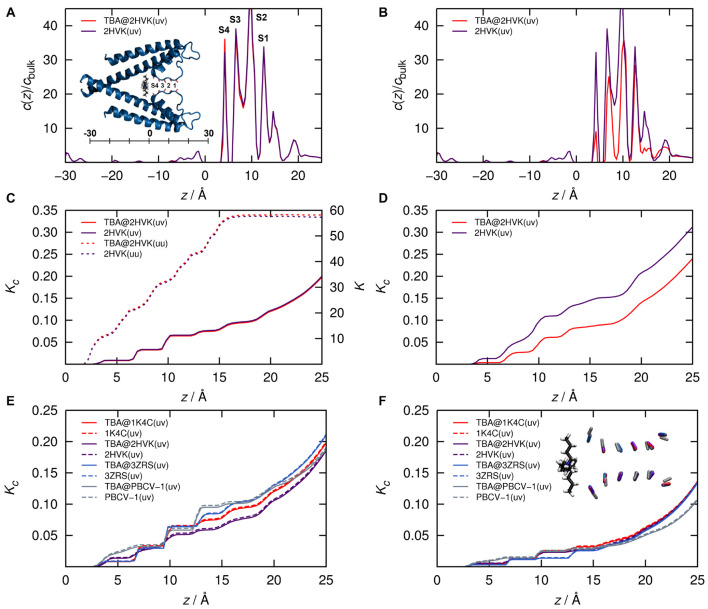
**(A,B)** K^+^ ion concentration profiles and **(C,D)** cumulative equilibrium constants as function of channel length coordinate z starting from the origin as shown in the inset in **(A)** from finite concentration (1 M, *uv*, solid lines) and infinite dilution (single hydrated ion, *uu*, dashed lines) 3D RISM calculations of KcsA (pdb code 2HVK) with (red) and without (purple) TBA placed in the cavity. **(E,F)** cumulative results for four reduced models with and without TBA comprising isolated carbonyl cages for KcsA (2HVK, Yohannan et al., 2007, crystalized with TBA and 1K4C (Zhou et al., 2001) crystalized without TBA), KirBac3.1 (3ZRS, Bavro et al., 2012) and the computationally determined filter model of Kcv_PBCV–1_ (Tayefeh et al., 2009; Hoffgaard et al., 2015). **(A,C,E)** from calculations with original force field, **(B,D,F)** with down-scaled filter carbonyl charges leading to halved local dipole moments. The inset in panel **(A)** represents the full 2HVK system in cartoon representation. The inset in panel **(F)** shows a superposition of all reduced filter carbonyl groups in the same color code as the curves.

For the calculation of *P*(S4) in the presence of the blocker, three rate constants are set to zero (crossed-out in red in Figure 3A). Binding and release of the K^+^ ion at the cytosolic side is no longer possible, since the blocker occupies the required place of the cavity ion (*k*_12_ *= k*_21_ = 0). With a similar rational also *k*_32_ = 0 because the ion in S4 in state 3 (*P*_3_) cannot leave into the cavity. In the KcsA structure (Faraldo-Gómez et al., 2007; Yohannan et al., 2007; Lenaeus et al., 2014) there is no space between S4 and the binding site of TPrA (see also inset in Figure 4A). The other rate constants in Figure 3A are assumed to be unchanged, as mentioned above and discussed in the Supplementary Material.

With these reasonable assumptions, the occupation probabilities of the states in the model in Figure 3A can be calculated from the rate constants of the open channel by Eq. S5.

The ion occupation probability of binding site S4 near the blocker includes all states with an ion in S4 resulting in:


(1) $$P\left(S4\right)=P_{3}+P_{4}+P_{5}$$

*P*_*m*_ is the probability of state *m* in Figure 3A and *P*(S4) the occupation probability of S4. These probabilities are shown in Figure 3B for the open unblocked channel and in Figure 3C for the blocked channel. State 2 is not occupied at all in the blocked channel, a trivial consequence of setting *k*_32_ and *k*_12_ to zero. Furthermore, the voltage dependence of *P*(S4) is drastically altered by the blocker (in contrast to the individual ion-hopping rate constants, except those being set to zero). This is because opening the cycle between *P*_1_ and *P*_2_ eliminates micro-reversibility. This does not matter at 0 mV because there is no net current. The inset in Figure 3C shows the values for the open and blocked channel taken from Figures 3B,C at 0 mV. The ion occupation probabilities *P*_3_, *P*_4_, and *P*_5_ are virtually unchanged by the blocker at 0 mV. Only the probability *P*_1_ is slightly increased since this state now has absorbed *P*_2_, but this does not affect *P*(S4). This is in line with the observation that filter population in crystal structures, and in our 3D RISM calculation, which are both without voltage, are unchanged by QA blockers (Faraldo-Gómez et al., 2007; Yohannan et al., 2007; Lenaeus et al., 2014).

Visual comparison of Figures 2B, 3C already suggests that the voltage dependence of $k_{\text{off}}^{\text{O}}$ and *P*(S4) are very similar. Their perfect coincidence (Figure 3D) corroborates the following model: After the blocker has bound, the ion distribution adopts a new steady or quasi-stationary state on the ns-time scale, since the transition time of a single ion is about 10 ns. This steady state lasts for an average time of 1/$k_{\text{off}}^{\text{O}}$ (about 3 μs) until the blocker no longer withstands the electrostatic repulsion and leaves the binding site.

It is obvious from the model of ion-hopping (Figure 3A) that the ion occupation of S2, *P*(S2), is identical to *P*(S4). In the following equations, we nevertheless use *P*(S4) because of two arguments: 1. S2 is further away from the binding site of the blocker. Thus, its contribution to the repulsive force is expected to be small 2. *P*(S2) and *P*(S4) have the same voltage dependency. Thus, replacing *P*(S4) by *P*(S4) + const^∗^*P*(S2) would merely lead to a slightly different constant pre-factor plus an additional fitting parameter in Eq. 3 without any influence on the core message of this investigation. It may be considered whether other K^+^ binding sites could have a similar effect as S2 and S4. Figure 3A shows that the alternatives *P*(S1) and *P*(S3) related to states 1 and 2. The probability of state 2 (*P*2) is zero and *P*1 has the opposite voltage dependency as $k_{\text{off}}^{\text{O}}$ (Figure 3C).

Therefore, the causal relationship between $k_{\text{off}}^{\text{O}}$ and *P*(S4) can be described by a weighted superposition of two different scenarios namely blocker release depending on the absence or presence of a K^+^ ion in S4. This leads to the following Eyring-type expression for the total rate constant in the form of:


(2) $$\begin{array}{c} k_{\text{off}}^{\text{O}}=k_{\mathrm{off},0}^{\text{O}}\left(1-P\left(S4\right)\right)\exp\left(-\Delta G_{0}/kT\right)\\ +k_{\mathrm{off},0}^{\text{O}}P\left(S4\right)\exp\left(-\Delta G_{\text{ion}}/kT\right) \end{array}$$

Here Δ*G*_0_ and Δ*G*_ion_ are the free energy barriers that the blocker needs to overcome in order to leave its binding site in the absence (Δ*G*_0_) or presence (Δ*G*_ion_) of an ion in S4, respectively; *k* and *T* have their usual thermodynamic meaning. Δ*G*_0_ and Δ*G*_ion_ are assumed to be independent of voltage. Voltage predominantly influences local ion occupancies, i.e., *P*(S4). The pre-factor $k_{\mathrm{off},0}^{\text{O}}$ could in principle depend on voltage or the voltage-dependent ion population for example via structural rearrangements at the inner mouth, which are energetically coupled to the filter configuration. However, we approximate the pre-factors with and without blocker as identical as they measure the effective curvature of the blocker free energy surface; the latter is governed by the channel structure and determines the intrinsic blocker dynamics. This should be largely unaffected when the overall structure remains essentially unaltered by the filter ions (Faraldo-Gómez et al., 2007); see also the similar discussion related to 3D RISM calculations below.

For our model, we also ignore the direct influence of the electric field on measured blocker dissociation. It has been shown that about 80-90% of the transmembrane voltage decays over the range of the selectivity filter (Jiang et al., 2002; Contreras et al., 2010; Andersson et al., 2018). This assumption is further corroborated by 3D RISM calculations (see below) that demonstrate that the energetic effect of typical electrostatic potential variations on singly charged ions are much smaller than the binding free energy change exerted by an ion placed at S4 on the blocker.

We therefore assume that all terms in Eq. 2 with exception of *P*(S4) are voltage-independent. They can be merged into constant factors, which simplifies Eq. 2 to the form used for fitting voltage dependency of the measured values of $k_{\text{off}}^{\text{O}}$ (Figure 3D):


(3) $$k_{\text{off}}^{\text{O}}=a\left(1-P\left(S4\right)\right)+cP\left(S4\right)=a+bP\left(S4\right)$$

This equation provides an excellent fit of $k_{\text{off}}^{\text{O}}$ in symmetric 100 mM KCl (Figure 3D) with the following fit parameters:


$$\begin{array}{c} a=k_{\mathrm{off},0}^{\text{O}}\exp\left(-\Delta G_{0}/kT\right)=31 400\pm 1 700s^{-1},\text{and}\\ b=c-a=k_{\mathrm{off},0}^{O}\left[\exp\left(-\Delta G_{\text{ion}}/kT\right)-\exp\left(-\Delta G_{0}/kT\right)\right]\\ \;=378 600\pm 8 600s^{-1}. \end{array}$$

This model equation is the principal result of the present work: It demonstrates that blocker release kinetics can be fully described by two system-specific parameters of ion/blocker interaction, *a* and *b*, or equivalently *a* and *c*. *a* and *c* represent the blocker dissociation rate constants for the two aforementioned scenarios where binding site S4 is empty or occupied, respectively.

To further test this causal relationship between *P*(S4) and $k_{\text{off}}^{\text{O}}$ the two parameters *a* and *b* were used for experiments with asymmetric KCl solutions. To this end, we measured $k_{\text{off}}^{\text{O}}$ of the TPrA block with 500 mM KCl on the external side and 100 mM KCl on the cytosolic side. Guided by the experience from measurements in symmetrical 100 mM KCl (Figure 2), we applied TPrA at a concentration of 5 mM. This assures blocking events at the critical range at negative voltages between −160 mV and −40 mV. These data cover the interesting region of the voltage dependency of $k_{\text{off}}^{\text{O}}$ which is necessary for a comparison with the data from symmetrical 100 mM KCl.

The orange curve in Figure 3D shows excellent agreement within the confidence bands between measured values of $k_{\text{off}}^{\text{O}}$ and those predicted from the ion hopping model with *P*(S4) for an external K^+^ concentration of 500 mM. It is important to emphasize that *a* and *b* were not fitted to the asymmetric data but taken from the results in symmetric solutions. *P*(S4) was calculated for 500 mM external K^+^ from the rate constants of ion hopping (Figure 3A) using the Supplementary Eqs. S5-S9.

The fact that the parameters *a* and *b*, determined for symmetrical KCl, also match the data from asymmetrical recordings confirms our model assumptions. The results in Figure 3D verify previous suggestions (Jara-Oseguera et al., 2007; Posson et al., 2013) that the rate constant of blocker release $k_{\text{off}}^{\text{O}}$ is determined by the electrostatic repulsion of the positively charged blocker by the K^+^ ions in site S4.

Since $k_{\mathrm{off},0}^{\text{O}}$ is unknown we can only estimate the relative change of the Eyring barrier, ΔΔ*G*, which is caused by the presence of the ion. The ΔΔ*G* value can be obtained from the relation between *a* and *c* of Eq. 4 and the coefficients in Eq. 3 resulting in:


(4) $$\Delta G_{\text{ion}}-\Delta G_{0}=\Delta \Delta G=-\ln\left(1+\frac{b}{a}\right)kT$$

With the values of *a* and *c* given above we can calculate an effective ΔΔ*G* = −2.6 ± 0.15 *kT*.

### Estimating the Influence of the Blocker on the Ion Distributions in the Selectivity Filter

A crucial assumption in the comparison between the voltage dependencies of $k_{\text{off}}^{\text{O}}$ and ion occupation in S4 is that the blocker does not influence the relevant rate constants of ion hopping in the model of Figure 3A. Thus, we determined the impact of the blocker on filter occupancies using the crystal structure of KcsA in complex with tetrabutylammonium (TBA, pdb code 2HVK, Yohannan et al., 2007; Figure 4A, inset). This corresponds to the “closed” KcsA structure, which at a first glance seems to be inadequate, since here we block the open Kcv_NTS_. However, in the “closed” KcsA channel only the bundle crossing gate is closed while the SF is open (Cuello et al., 2017), and that is exactly what we need to represent the Kcv_NTS_. Because of the absence of a cytosolic gate in Kcv_NTS_ (Rauh et al., 2017b) the closed KcsA bundle crossing is of no concern as we only deal with the SF. Thus, the KcsA structure is a valid model system for interpreting the experimental data from Kcv_NTS_ in a structure/function context due to the similar SF and overall pore architecture of both channels.

The KcsA template structure was exposed to aqueous KCl solutions for 3D RISM calculations (Kast et al., 2011) in the presence and absence of blocker without changing the structure. This is in line with the apparent insensitivity of the pore structure to the blocker (Guo and Lu, 2001; Jara-Oseguera et al., 2007; Lenaeus et al., 2014). 3D RISM theory (Beglov and Roux, 1996, 1997; Kovalenko and Hirata, 1998) yields the approximate equilibrium distribution of solvent atoms and ions around (and inside) a molecule, from which ion occupancy measures can be computed in two different ways: a partitioning constant *K*_*c*_ (mass action law) and a thermodynamic binding constant *K* at infinite dilution. Details are discussed in the Supplementary Material.

Results for calculations with and without TBA (the latter obtained by removing TBA coordinates from the pdb structure) are shown along with a cartoon of the channel model in Figure 4A. The results with the original force field charges clearly corroborate the underlying hypothesis: The population of filter K^+^ ions (in local concentration and the cumulative scale) and in both the finite concentration and infinite dilution setup (Figures 4A,C), are largely unaffected by TBA in its blocking site.

The situation only changes as soon as we (unphysically) scale the charges of the filter carbonyl ions to exhibit a local dipole moment of 50% of the original (Figures 4B,D): Only under this artificial condition the absolute filter population of K^+^ ions decreases after placing TBA close to the exit of the SF. This indicates that the SF is remarkable in the context of protein/K^+^ ion interactions; its architecture and electrostatic energy landscape is responsible for the apparent population decoupling. In other words, the ions in the filter do not “feel” the presence of a blocker ion.

To test whether this finding from the KcsA structure can be extrapolated to the experimental data from Kcv_NTS_ in particular, and all K^+^ channels with this type of SF in general we also calculated the blocker effect on K^+^ ion occupancies in maximally reduced channel models. These contain only the isolated carbonyl groups of the SF; all atoms representing the protein background are removed. Analogous calculations as in Figure 4A were done for two reduced template structures of KcsA (2HVK and 1K4C) as well as on two further isolated filters from KirBac and Kcv_PBCV–1_ (Figures 4E,F). TBA was placed in the same position relative to the SF, which corresponds essentially to the closest possible distance between blocker and filter. In this way, we examined the upper limit of the blocker effect on the filter ions. Results for full carbonyl charges and corresponding halved dipole moments are shown in the bottom row of Figure 4. Notably, despite modest structural diversity of the model filters (see inset in Figure 4F), the calculations confirm the independence of the occupation of K^+^ in the SF on the presence of the blocker. Worth noting is that the relative effect of halving the dipole moments is, different from the results in the full protein, practically negligible in the isolated carbonyl cages. This means that the protein environment actually counteracts the strongly attractive carbonyl forces exerted on the K^+^ ions. Conversely, this result further emphasizes the dominant role of the filter carbonyl geometry and electrostatics for controlling the K^+^ occupation probability. The data again confirm our basic premise that blocker kinetics can be derived from equilibrium ion concentrations determined from the blocker-free channel pore.

For the preceding analysis of the functional data in the context of the filter structure it is important to note that the ion concentration profile can be interpreted as the effective single-ion free energy landscape in the mean field of all ions, which are on average present in a structurally averaged SF. This energy landscape, which is seen by the ions, hence determines the rate constants of ion hopping. Therefore, when the concentration profile is unchanged, we can assume that the hopping kinetics are also unchanged (except of course for those transitions that are made impossible by the blocker). In this context it is plausible to assume that quasi-stationary concentration profiles at a given voltage will remain unaffected by the blocker even under an external potential due to the strong electrostatic carbonyl-ion interactions, though we are not able to quantitatively model such a system on the basis of an equilibrium theory such as 3D RISM.

Furthermore, 3D RISM calculations allow for an analytically computable estimation of the excess chemical potential, *μ*^ex^, of molecular structures in solution (Kast and Kloss, 2008; Tielker et al., 2018, 2021), which can be used to check model assumptions. One interesting case is to study the impact of an explicitly placed K^+^ ion at position S4 in the reference KcsA channel structure. Results for the isolated protein, channel with K^+^ at S4, channel with TBA, and channel with both, K^+^ and TBA are −16925.01 (KcsA), −16580.38 (KcsA-K^+^), −16920.37 (KcsA-TBA), −16747.73 (KcsA-TBA-K^+^) kJ/mol, respectively. Together with the direct TBA-K^+^ interaction energy taken from the force field of Δ*E* = 243.34 kJ/mol we obtain for an approximation to the relative blocker release free energy between K^+^-loaded and free structure a value of:


(5) $$\begin{array}{c} \mu^{\text{ex}}\left(\text{KcsA}-{\text{K}}^{+}\right)-\mu^{\text{ex}}\left(\text{KcsA}-\text{TBA}-{\text{K}}^{+}\right)\\ -\left[\mu^{\text{ex}}\left(\text{KcsA}\right)-\mu^{\text{ex}}\left(\text{KcsA}-\text{TBA}\right)\right]-\text{Δ}E\\ =-71.34\mathrm{kJ}/\text{mol} \end{array}$$

Although we ignored thermal fluctuations in this simple model, rigid body cancelation allows at least for estimating the order of magnitude of the explicit ion effect. Clearly, the presence of an explicit ion strongly disfavors blocker binding. This energetic effect is much larger than that from external voltage. Even if we assume a 100 mV drop over the distance between S4 and blocker site (which is in practice much smaller), this would amount to only roughly 9.6 kJ/mol, i.e., a value much smaller than the direct interaction effect. This corroborates the assumption that a direct influence of membrane voltage on the blocker can be ignored for our model in Eqs. 2, 3.

Given the magnitude of the observed free energy change, one might ask how the relatively small ion modulation of the relative barriers of ΔΔ*G* = −2.6 *kT* (ca. –6.4 kJ/mol) from Eq. 4 comes about. Here, we have to consider that an explicit ion lifts both, the absolute magnitudes of free energies in the bound minima and the transition states. The small ΔΔ*G* therefore implies the change of energetic *distance* between transition and bound states. This merely means that the transition state feels less repulsive interaction with the S4 ion than the bound state. Mechanistically, this implies that the transition state is located farther away from the S4 ion than the bound state, which is of course plausible.

## Conclusion

The experimental results culminating in Figure 3D as well as the calculations in Figure 4 have important consequences for understanding the mutual interactions between blocker and channel proteins: The excellent fit verifies in quantitative terms the assumption (Heginbotham and Kutluay, 2004; Jara-Oseguera et al., 2007; Posson et al., 2013) that an ion close to the bound blocker is responsible for releasing the latter from its binding site. Our data now identify the K^+^ ion in binding site S4 (possibly with a smaller contribution by the S2 ion) in the selectivity filter as the relevant repulsing ion. The occupation of this site determines in quantitative terms blocker dissociation.

The good agreement between experimental kinetic analysis in the Kcv_NTS_ channel and calculations on the structurally similar channel KcsA, which was crystallized in the presence of a blocker, reveals a surprising feature at the atomic level: Even though the electrostatic interaction/repulsion between blocker and K^+^ ions is symmetric, the strong affinity between the filter carbonyl groups and K^+^ ions basically “shields” the latter in a kinetic sense from any impact by the blocker ion even at close proximity by preserving filter ion occupancies. The population dynamics in the filter is therefore a consequence of the protein environment, and dominantly controlled by the specific ion-carbonyl interactions alone.

As a signature of this phenomenon, the occupation probability of the binding site in the SF in vicinity to the blocker (*P*(S4), Eq. 2) can be correctly determined from the rate constants of ion hopping (*k*_*ij*_ in Figure 3A). The latter were obtained from experimental data of the open channel (Rauh et al., 2018), using a global fit of IV curves and the voltage dependence of the rate constant of channel closing.

All these results corroborate the power of using excess noise analysis of electrophysiological recordings together with model-based analysis (Rauh et al., 2018) to test predictions from structural data and computational modeling. The fact that SF populations are unaffected by the blocker ion, irrespective of the exact filter geometry and the molecular features of the remaining protein, prompt further-reaching speculations: a direct consequence of these results is that functional data measured in the absence of the blocker and the respective rate constants suffice as input for quantifying the population variable in the kinetic release model. The presence of the blocker can then be simply modeled by setting rate constants of impossible pathways to zero, leaving only two adjustable parameters that completely describe the blocker release under diverse bath conditions. Because of the conserved pore architecture, it is tempting to predict that these features are transferable to other K^+^ channels. This can be tested in future work, along with a development of a similar model for blocker binding, to close the gap to a general quantitative blocking/unblocking model approach, that will be helpful in rational drug design for K^+^ channel targets.

## Data Availability Statement

The software for the 3D RISM calculations has been developed in our laboratory and can be made available for collaboration purposes on request. The program “bownhill” for extended beta distribution analysis as well as the source code is available on request from the corresponding author.

## Author Contributions

TG, TW, ND, and OR performed the experiments. MU performed the 3D RISM calculations. GT designed the research. SMK designed the research, analyzed the 3D RISM results, and wrote the manuscript. U-PH and IS designed the research, analyzed the data, and wrote the manuscript. All authors contributed to the article and approved the submitted version.

## Conflict of Interest

The authors declare that the research was conducted in the absence of any commercial or financial relationships that could be construed as a potential conflict of interest.

## Publisher’s Note

All claims expressed in this article are solely those of the authors and do not necessarily represent those of their affiliated organizations, or those of the publisher, the editors and the reviewers. Any product that may be evaluated in this article, or claim that may be made by its manufacturer, is not guaranteed or endorsed by the publisher.
