# Supplementary material for: Asymmetric Interplay Between K+ and Blocker and Atomistic Parameters From Physiological Experiments Quantify K+ Channel Blocker Release
Source: Front Physiol. 2021 Oct 29;12:737834. doi: 10.3389/fphys.2021.737834 (PMC8586521; doi:10.3389/fphys.2021.737834)
Supplement: Supplementary file 1 [file Presentation_1.pdf]

frontiers

Supplementary Material to

Asymmetric interplay between K^+ and blocker and atomistic parameters from physiological experiments quantify K^+ channel blocker release

Tobias S. Gabriel[1], Ulf-Peter Hansen[2], Martin Urban[3], Nils Drexler[4], Tobias Winterstein[1], Oliver Rauh[1], Gerhard Thiel[1], Stefan M. Kast[3] and Indra Schroeder[1,4*]

[1]Plant Membrane Biophysics, Technische Universität Darmstadt, 65287 Darmstadt, Germany
[2]Department of Structural Biology, Christian-Albrechts-Universität zu Kiel, 24118 Kiel, Germany
[3]Physikalische Chemie III, Technische Universität Dortmund, 44227 Dortmund, Germany
[4]Physiology II, University Hospital Jena, Friedrich Schiller University Jena, 07743 Jena, Germany

*Correspondence should be addressed to Indra Schroeder Physiology II, University Hospital Jena, Friedrich Schiller University Jena, 07743 Jena, Germany, indra.schroeder@med.uni-jena.de

Published in Frontiers in Physiology, 2021, doi: 10.3389/fphys.2021.737834

Amplitude histograms used for the determination of the rate constant of fast gating and fast blocking

Amplitude histograms from control experiments are compared with those obtained with 0.1 mM cytosolic TPrA at +140 mV (Fig. S1A) and with 5 mM cytosolic TPrA at -140 mV (Fig. S1B). The direct determination of the single-channel current I_{true} (vertical dashed lines) from the time series in Fig. 1 would require the measurement with an (non-existing) amplifier of sufficient temporal resolution and very low high frequency noise. However, I_{true} can be determined by analyzing the amplitude histograms with extended beta distributions[1–4], as described in Materials and Methods. I_{true} is not significantly different with or without TPrA.

At positive voltages, the apparent open peaks (A, A') are well separated from the closed peak, i.e. the baseline (C) at 0 pA (Fig. S1A). TPrA leads to a strong broadening of the apparent open peak, as compared to the control experiment indicating the presence of many unresolved blocking events[1,5]. Differences in the width of the closed peak result from different baseline noise (e.g. 0.4 pA or 0.5 pA with and without TPrA in Fig. S1A) and are not an effect of the blocker. At higher negative voltages, the "valley" between the apparent open peak and the closed peak is filled up by the strong intrinsic sub-millisecond gating (state M in Fig. 2A) already in the control experiments[2,3]. The short blocking events by TPrA deform the open-channel distribution even further. In either case, a maximum defining the position of the apparent open peak is no longer visible, and the analysis by extended beta distributions has to be applied.

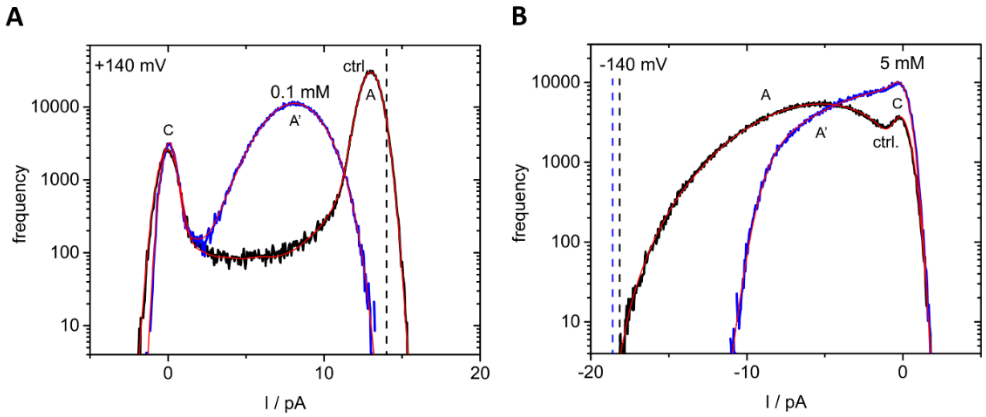

Supplementary Figure S1. Examples for the differences in amplitude histograms obtained in the absence (black) and presence of TPrA (blue). The red lines show fits of the amplitude histograms by extended beta distributions with the Markov model in Fig. 2A as described in Materials and Methods. **(A)** 0.1 mM TPrA at +140 mV **(B)** 5 mM TPrA at -140 mV. The "true" single-channel currents obtained from the fits (with and without TPrA) are indicated by vertical dashed lines. In (A), these two lines overlap. The slight difference in I_{true} with and without TPrA in (B) is due to experimental variance and fitting scatter. The closed (C) and the apparent open channel peaks (A and A' for apparent current without and with TPrA, respectively) are labelled.

The reliability of the fits determining the rate constants of TPrA block

In the case of the 4-state model without TPrA, there is a clear relationship between the gating processes (Fig. 2A) and the characteristics of the amplitude histogram (Fig. S1)[1,2]. Briefly, O-S gating (Fig. 2A, S = slow) contributes to the depth of the valley between open and closed peak in Figure S1. O-M gating (Fig. 2A, M = medium) shapes the slope between open and closed peak. O-F gating (Fig. 2A, F = fast) contributes to the width of the apparent open peak. O-F and O-M gating cause the difference between the true (I_{true}) and the apparent (I_{app}, A in Fig. S1) single-channel current.

Clear characteristic features in the amplitude histograms as found for the intrinsic gating in the absence of blockers cannot be attributed to the blocking process O-B. At positive voltages, the rate constants of channel opening by blocker dissociation ($k_{BO} = k_{off}^{O} = 1/\tau_{blocked}$ = inverse dwell time in the blocked state) come close to those of the O-M gating (k_{MO}) as indicated by the blue circles approaching the blue and black diamonds in Fig. S2A. At negative voltages, k_{off}^{O} (orange circles) comes close to k_{FO} (black triangles). Thus, it would be expected that the numerical evaluation would be difficult. However, it is reliable because of favorite numerical conditions.

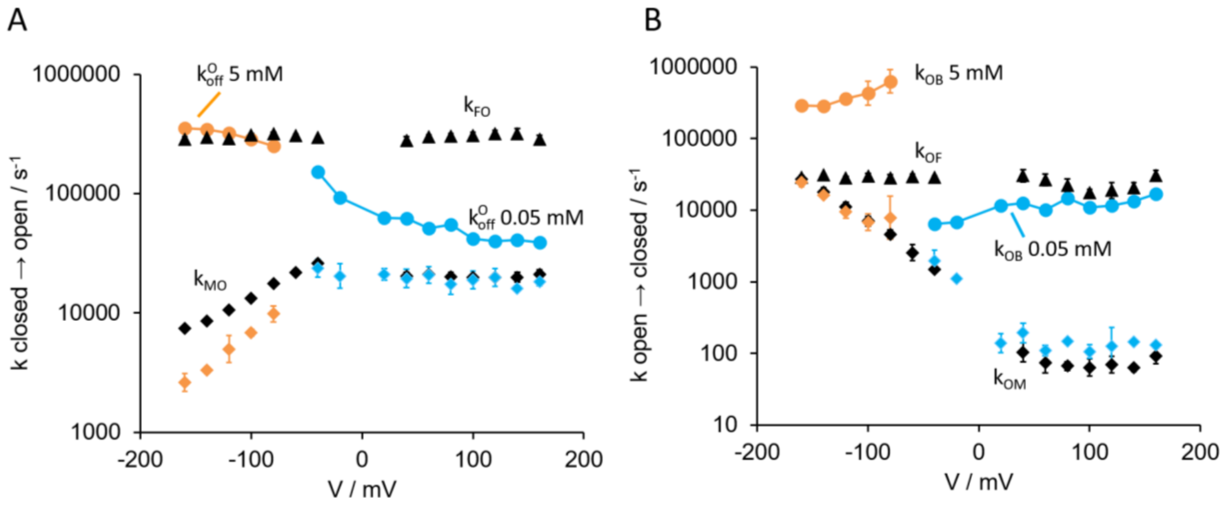

Supplementary Figure S2. (A) Comparison of rate constants of channel opening by blocker release ($k_{\mathrm{BO}} = k_{\mathrm{off}}^{\mathrm{O}}$) and medium (k_{MO}) and fast (k_{FO}) intrinsic gating. **(B)** Comparison of the rate constants of channel closing by blocking (k_{OB}) and during medium (k_{OM}) and fast (k_{OF}) intrinsic gating. Blocking: blue (0.05 mM TPrA) and orange (5 mM) filled circles. O-M gating without TPrA: black diamonds, 0.05 mM TPrA: blue diamonds, 5 mM TPrA: orange diamonds. O-F gating without TPrA: black triangles. Data points are geometric means obtained from three to four different channels. The error bars give the geometric standard deviation.

At positive voltages, state B could possibly be confused with state M because of the similar dwell times. However, the rate constant of blocking k_{OB} (blue circles in Fig. S2B) is by far higher than that of k_{OM} (blue and black diamonds) even at low TPrA concentrations. This leads to dominance of B over M, making its determination insensitive to interference by the O-M gating. At negative voltages, only higher concentrations were analyzed. Here k_{OB} is much faster even than k_{OF} (orange circles in Fig. S2B). Under these conditions, the rate constants of blocking override those of the intrinsic gating. This makes the determination of the rate constants of blocking reliable, but those of O-F gating unreliable. Thus only the values of the O-F gating without TPrA are shown (black triangles in Fig. S2A,B). O-F gating is not of interest here, and any error in its determination would not significantly affect the values of the rate constants of blocking. In contrast, the error sum of the fits was still slightly sensitive to the values of the O-M gating (diamonds in Fig. S2A,B). However, even in this case, any error in the O-M rate constants would not significantly affect the values of blocking because k_{OB} is much faster than k_{OM} at both negative and positive voltages (for those concentrations analyzed in the respective voltage ranges).

However, two effects limit the range where the rate constants of blocking can be determined with high accuracy. First, due to their voltage dependence, k_{OB} and k_{OM} at 0.05 mM TPrA (blue) meet each other at about -80 mV. At more negative voltages, k_{OM} starts to override k_{OB}. However, the range of determination of blocker kinetics is extended with increasing blocker concentration, which increases k_{OB} (Fig. 2C), thus moving the point of intersection to more negative potentials. Consequently, the curve of the concentration-independent $k_{\mathrm{off}}^{\mathrm{O}}$ (Fig. 2B) could be composed from the results obtained at different TPrA concentrations.

Second, high blocker concentrations decrease the amplitude of the apparent single-channel current (Fig. S1B). This lowers the signal-to-noise ratio (the limiting factor for the analysis by extended beta distributions[1]) as it merges the characteristic features of the amplitude histograms. Because of the voltage dependence of blocking (Fig. 2B,C), this causes a limit with increasing positive voltages, as indicated by the orange circles (5 mM TPrA) in Fig. S2A,B, ending at -80 mV.

The rate constants of the intrinsic gating in the presence of TPrA

The rate constants of the intrinsic gating of the Kcv_{NTS} channel without TPrA are those of the 4-state sub model consisting of states O, F, M and S in Fig. 2A. They have already been determined at various cytosolic and external K^+ concentrations in the range of -160 mV to +160 mV[3]. Here, they are not of interest for the kinetics of blocking. Nevertheless, it may be interesting, whether the blocker also influences the single-channel current or the rate constants of the intrinsic gating.

There is no influence of the blocker on I_{true} (Fig. S3A), the true open-channel current. This is the hypothetical open-channel current obtained from sojourns in the open state, which are not interrupted by gating or blocking events. It can be determined from extended beta distribution analysis as in Fig. S1[1].

As mentioned above, the O-F gating cannot be determined with high accuracy in the presence of the blocker. Nevertheless, in the presence of TPrA, a change of these rate constants by a factor 2 to 3 decreased the quality of the fits. Thus, it can be assumed that these rate constants are also nearly voltage-independent like the control values in Fig. S2. Their exact values are not of interest here, and their choice does not influence the values of the rate constants of blocking. However, as found previously (e.g. ref.[6]), their ratio is much more reliable than their individual values. The current I_{OF} is calculated by means of the ratio

$$I_{OF} = I_{true} \cdot \frac{k_{FO}}{k_{FO} + k_{OF}} . \tag{S1}$$

I_{OF} is the current that could be recorded with a setup that can resolve O-S and O-M gating, but not O-F gating. This was in a previous work achieved in high K^+ concentrations with the VC100 low-noise, high bandwidth patch-clamp amplifier of Chimera Instruments, LLC, New York, NY, USA[2]. Figure S3B shows that I_{OF} is unchanged by TPrA. This is an indication that the O-F gating is not influenced by the blocker.

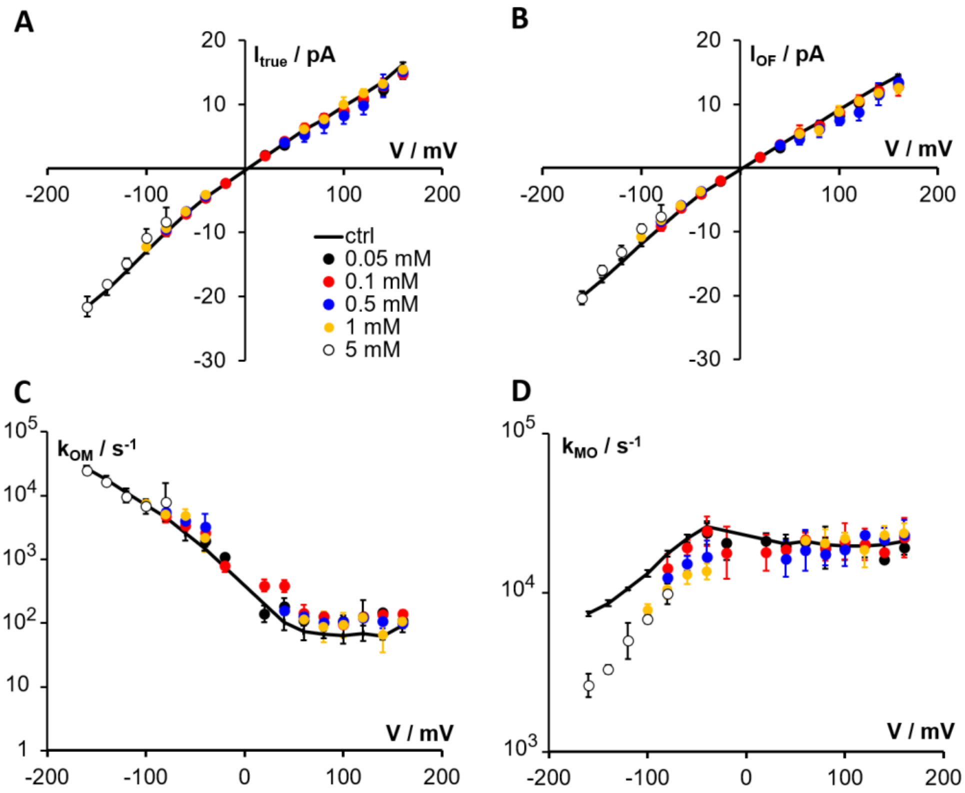

Supplementary Figure S3. Influence of cytosolic TPrA on **(A)** the true single-channel current I_{true}, **(B)** the current I_{OF} attenuated by the O-F gating (Eq. S1) and the rate constants **(C)** k_{OM} and **(D)** k_{MO} of the O-M gating (See Markov model in Fig. 2A). Control values without TPrA (black line) were taken from ref.[3]. Color-coding for the TPrA concentrations is the same in all four panels. (A and B: Mean and standard deviation, B and C: Geometric mean and geometric standard deviation. Three to four individual channels per data point.).

The values of k_{OM} (Figs. S2B and S3C) are not significantly different from those found without blocker. We have previously identified k_{OM} as the rate constant of the closure of the selectivity filter, which is influenced by the ion occupation of the filter binding sites[3]. However, the reverse reaction k_{MO} at negative voltages is decreased by the blocker leading to a stronger voltage dependence (Fig. S3D). Since the mechanism of k_{MO} is currently still unknown, we will not speculate here on the mechanistic details of the influence of the blocker on k_{MO}.

The O-S gating showed higher values of the rate constants in the presence of TPrA. However, these rate constants are too slow for a meaningful evaluation by fitting amplitude histograms, so we refrain from showing or interpreting them. For the more adequate dwell time analysis, the measured time series are too short. Similarly, any statements regarding the O-S rate constants were also avoided in the previous study without blocker[3].

Binding of the blocker

Since only $k_{\text{off}}^{\text{O}}$ is in the focus of this investigation, the discussion of k_{OB} (Fig. 2C,D) is given here. The rate constant of blocker binding, k_{OB}, increases with the TPrA concentration (Fig. 2C). The *a-priori* expectation that k_{OB} is proportional to the concentration of TPrA is verified in Fig. 2D by the coincidence of k_{on}^{O} at different concentrations of TPrA: k_{on}^{O} is k_{OB} at 1 M TPrA. According to the relation

$$k_{\text{OB}} = k_{\text{on}}^{\text{O}}[\text{TPrA}] \tag{S2}$$

k_{on}^{O} is calculated by dividing k_{OB} by the TPrA concentration. The voltage dependence of k_{on}^{O} (Fig. 2D) is much weaker than that of $k_{\text{off}}^{\text{O}}$ (Fig. 2B) and can be fitted by a linear model (black line in Fig. 2D).

$$k_{\text{on}}^{\text{O}} = k_{\text{on},0}^{\text{O}} + s \cdot V \tag{S3}$$

with V as membrane voltage in mV, k_{on}^{O} = $1.67 \cdot 10^{8} \pm 1.1 \cdot 10^{7}$ s^{-1} mol^{-1} and $s = 6.6 \cdot 10^{5} \pm 1.8 \cdot 10^{5}$ mV^{-1} s^{-1} mol^{-1}. The coincidence of k_{on}^{O} obtained from TPrA concentrations varying by a factor of 100 (0.05 mM to 5 mM) emphasizes the linear relationship between k_{OB} and blocker concentration. Furthermore, it demonstrates the reliability of the extended beta distribution analysis for the determination of the rate constants of blocking and the applicability of the simplified Markov model in Fig. 2A. The linear relationship between k_{on}^{O} and voltage V in Eq. S3 may indicate that the voltage dependence of blocker association results from coupling to the stream of ions.

Evidence for the applicability of the Markov Model in Fig. 2A for evaluating the rate constant $k_{BO} = k_{off}^{O}$ of blocker dissociation from the measured time series

In the Markov model in Fig. 2A, the binding and dissociation of the blocker can occur only when the system is in the open state O, which is shared for the intrinsic O-F, O-M and O-S gating and the blocking. More correct would be a system with two parallel Markov models (Fig. S4A), namely the model for the intrinsic gating and the two-state model D-B (blocker dissociated and blocked/bound). The D-B model may even have different rate constants depending on the states of the intrinsic gating. However, blocking events that occur completely during a closed event of the intrinsic gating are unobservable and thus do not need to be modelled. In the Markov model of Fig. S4A, the measured open state would occur when both sub-models are in the open state (i.e. O and D), a process identical to that in the real channel. As mentioned in the main text, the usage of the simplified model may lead to errors in the determination of the rate constants by the fitting routine.

The scenario in Fig. S4B applies to the situation at high negative voltages, where the open state of the O-F (and of the O-M gating) (Fig. S2B) gating is much longer (about 40 µs) than the open and closed states of blocking ($1/k_{OB}$ and $1/k_{BO}$ ca. 3 µs), and the closed state F being of similar duration ($1/k_{FO}$ ca. 3 µs) as the blocked state B. The black line gives the open-closed transitions as caused by O-F gating in the real channel (model in Fig. S4A). The blue line represents the blocking, the measured current is drawn in red.

For simplicity, all blocking events are shown with dwell times of equal length representing the related inverse average rate constant k_{av} in the dissociated state (D) and the blocked state (B) for the clear identification of the events. In reality (and in the fitting routine), the durations are determined by a random generator selecting the actual time of a transition from the exponential function of the probability of that transition calculated for the average rate constant k_{av}. In the resulting amplitude histogram, the average of thousands of transitions are included leading to the average k_{av}[7].

The errors in determining k_{BO} caused by the use of the model in Fig. 2A instead of the correct one in Fig. S4A are discussed now. The difference between the two models results from the fact that the model in Fig. 2A can create open-close transitions only from the open state. Thus, it generates a series of sojourns in the states B, F, M, S without interference by other states in contrast to those occurring at the transitions of the O-F gating in the red trace of Fig. S4B, labels (a) and (b). The durations in the closed (blocked) states (model of Fig. 2A) are determined by the individual rate constants k_{BO}, k_{FO}, k_{MO} and k_{SO}. However, the life time of open events is given by $1/k_{close} = 1/(k_{OB} + k_{OF} + k_{MO} + k_{OS})$[7,8]. k_{OB}, k_{OF} and k_{OM} are additionally determined in the fit by the number of transitions into the B, F or M state. At -160 mV, the ratios $r_{FB} = k_{OF}/k_{OB}$ and $r_{MB} = k_{OM}/k_{OB}$ are less than 0.1, and k_{OS} is negligible. In order to model the measured blocking kinetics correctly, the fit has to use a $k_{OB,fit} = k_{OB} - k_{OF} - k_{OM}$, i.e. approximately 0.8 k_{OB}. For the present analysis, this is of no consequence, because we are interested only in $k_{BO,}$ the dissociation in the open state.

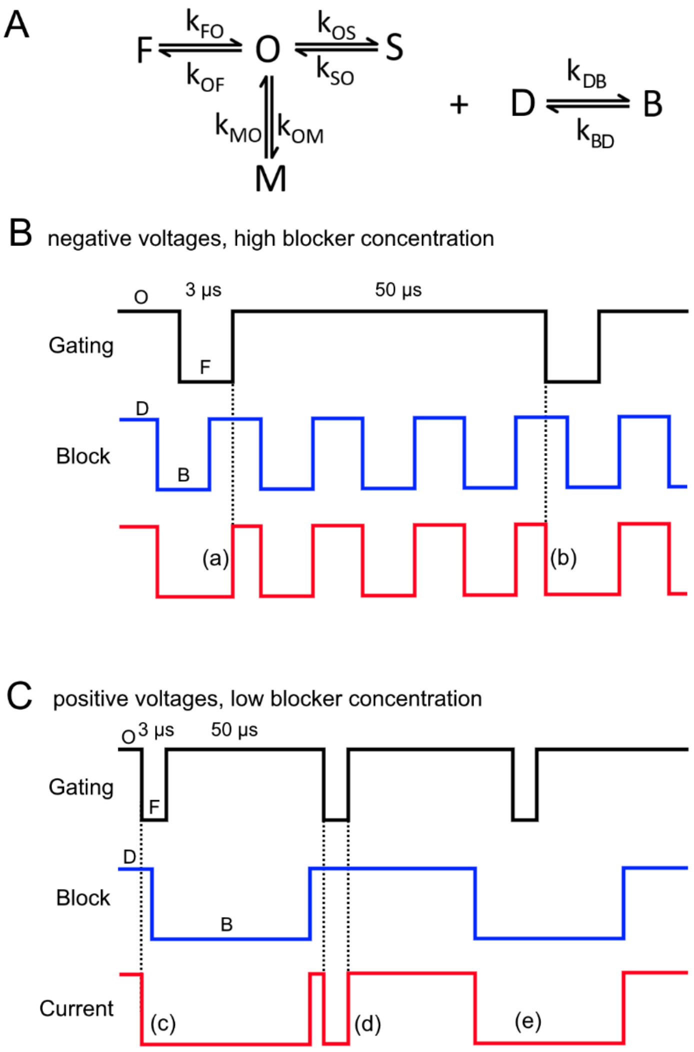

Supplementary Figure S4. Interference of blocking with intrinsic gating. **(A)** Separate Markov models for intrinsic gating (O, F, M, S) and blocking (D, B). Current can only flow when D and O coincide. **(B, C)** Different scenarios for the discussion of the effect of using the model in Fig. 2A instead of the model in (A). The lines give the schematic time courses (without lowpass filter and noise), which are generated by the model in (A): black: O-F gating, blue: blocking, red: measured current. (B) Interference of O-F gating (black) and blocking (blue) as occurring in the experiments at high negative and (C) at positive and low negative voltages. How good this can be reproduced by the model in Fig. 2A is discussed in the text. Labels (a) – (e) are referred to in the text.

k_{BO} is unaffected by the choice of model when blocking occurs completely within the open time of O-F or O-M gating (label (d)). A problem may arise at the edges of an open event of the intrinsic gating as shown in the red trace of Fig. S4B when in the measurement blocking events and O-F gating overlap (labels (a) and (b)). O-M gating is not discussed here, because the durations in the closed state M are much longer than those of B and thus the related events will not influence the fitted rate constants of blocking because they are assigned to M.

At the O-F transitions in the real system (Fig. S4B, black trace), three possible scenarios have to be discussed. On the left-hand side (label (a)), the transition of the dissociation of the blocker coincides with the F state. The apparent dwell time in B is prolonged, on average by 1.5 µs (half dwell time in F) as compared to 3 µs (average dwell time in B). This has a negligible effect on the determination of k_{BO}, because the chance that this occurs (k_{OF}/k_{OB}) is small. On the right-hand side (label (b)), blocking (O-B transition) occurs during the F state. Again, the apparent blocked state is prolonged, and this sojourn in state F is lost. The shortened open events next to the prolonged B-states may have a minor influence on the averages value of the open time $1/k_{av}$ considered above. As a summary, it can be stated that because of the numerical situation the influence of the effects at the O-F transitions on the determination of k_{BO} can be ignored, since they are not larger than the fitting scatter.

At positive and low negative voltages, the rate constants of blocking become slower and are in the same range as k_{OF} (Fig. S2). At these voltages, also the blocking events are of similar length as the dwell time in the open state of the O-F gating (Fig. S4C). Very similar scenarios as in Fig. S4B have to be considered here. The red trace shows the situation that blocking occurs during state F (label (c)). As in the red trace of Fig. S2B, the dwell time in state B is prolonged. It is still expected that it rarely occurs that B just coincides with the O-F transition (label (c)). More often is the occurrence of state F during the open (dissociated) or closed (associated) state of blocking (labelled by (d) and (e) in Fig, S4C). This has no effect on the dwell time in B. The effects on the dwell time in the open state are not of interest here, because we are interested only in k_{BO}. In addition, the relative prolongation of the individual sojourn in B in case (d) is very small. On average, it is $1/k_{BO,fit} = 1/k_{BO} + ½\, 1/k_{FO}$ about 25 µs + 1.5 µs. The same holds for a scenario corresponding to Fig. S2B (a) where the blocker dissociation occurs during state F (not shown) and consequently the apparent sojourn in B is prolonged. The interference with O-M gating can be ignored because k_{OM} is so small (Fig. S2B) that these events are extremely rare. Thus, Figs. S4B and S4C show that fitting on the basis of the model in Fig. 2A yields reliable results for k_{BO}.

The rate constants of ion hopping, which are not affected by the presence of the blocker at 0 mV, are also not affected in the presence of transmembrane voltage.

The evidence for the absence of an effect of the blocker on the ion distribution from crystal structure analysis cited in the main text and by the 3D RISM calculations is obtained for a membrane voltage of 0 mV. The following arguments underpin that this conclusion also holds under conditions of a membrane voltage: The rate constants k_{ij} in Fig. 3A are a constant term $k_{ij,0}$, which is multiplied by an exponential term in the case of the voltage-dependent rate constants $k_{ij} = k_{ij,0} \cdot \exp(sV / V_{ij})$ (see also Eqs. S8 and S9, below).

Thus, the ion occupation probabilities in the filter are completely determined by $k_{ij,0}$ and the exponential term. The $k_{ij,0}$ are unaffected by the blocker, an assumption which is confirmed by unchanged ion occupations in the crystal structures[19,22,23] (at 0 mV) and the 3D RISM calculations.

This causes the occupation probabilities predicted by the flux model in Fig. 3A being unaffected by the blocker at 0 mV (inset in Fig. 3C), except that the "forbidden" state 2 is now absorbed by state 1.

The other question regarding the exponential term is whether the Eyring barriers imposing the voltage dependency represented by *s* and V_{ij} are modified by the blocker. The basic message is that several mutations of the channel protein in the cavity and mutations affecting the anchoring of the selectivity filter do not modify the voltage dependency of the sub-millisecond channel closure[21,25] (k_{OM}, Fig. S3C). Since k_{OM} is determined by the ion occupations of states 3 and state 4 in the selectivity filter (Fig. 3A, and ref.[21]), this indicates that the selectivity filter is so stable that external influences cannot change the voltage-dependent exponential terms of the rate constants of ion hopping.

Calculation of flux rates and occupation probabilities from the flux model

The cyclic model of ion transport in Fig. 3A has five different states of ion occupancy (clockwise numbered from 1 to 5). The open-channel current can be calculated from the rate constants k_{ij} between these states. (The indices of the rate constants of the ion hopping model are given as numbers or by *i,j* if not specified, those of the gating process (Fig. 2A) by letters, excluding *i,j*). Clockwise motion in the model represents outward flux, anticlockwise motion equals inward flux; the net single-channel current is the difference of the two fluxes. The equation for the fitting of measured IV curves in the previous work[3] has been set up with the so-called "arrow scheme"[9]. This algorithm enables an easy generation of flux equations

$$I = \text{outward current - inward current} = e\frac{\overset{1\;\;2\;\;3\;\;4\;\;5\;\;1}{\left[\rightarrow\rightarrow\rightarrow\rightarrow\rightarrow\right]} - \overset{1\;\;2\;\;3\;\;4\;\;5\;\;1}{\left[\leftarrow\leftarrow\leftarrow\leftarrow\leftarrow\right]}}{D_1^{C5}+D_2^{C5}+D_3^{C5}+D_4^{C5}+D_5^{C5}} \tag{S4}$$

and of the occupation probabilities (*m* = 1, 2, 3, 4, 5)

$$P_m = \frac{D_m^{C5}}{D_1^{C5}+D_2^{C5}+D_3^{C5}+D_4^{C5}+D_5^{C5}}\,. \tag{S5}$$

e is the unit charge, the numbers 1-5 represent the states in the ion-hopping model (Fig. 4A). An arrow between the numbers *i* and *j* stands for the rate constant k_{ij} or k_{ji} depending on the direction of the arrow. Each row in square brackets gives the product of the rate constants. The matrices D_i^{C5} (C5 means "cyclic 5-state model") are defined as follows:

$$D_1^{C5} = \overset{1\;\;2\;\;3\;\;4\;\;5\;\;1}{\begin{bmatrix} & \rightarrow & \rightarrow & \rightarrow & \rightarrow \\ \leftarrow & & \rightarrow & \rightarrow & \rightarrow \\ \leftarrow & \leftarrow & & \rightarrow & \rightarrow \\ \leftarrow & \leftarrow & \leftarrow & & \rightarrow \\ \leftarrow & \leftarrow & \leftarrow & \leftarrow & \end{bmatrix}} = \sum_{m=1}^{5}\prod_{i=2}^{m} k_{i,i-1}\prod_{j=m+1}^{5} k_{j,j+1}\,. \tag{S6}$$

The rate constants in a row represented by an arrow are multiplied, and the products of each row are added. The indices are cyclic, i.e., the index following 5 is not 6, but 1. For the matrices D_2^{C5} to D_5^{C5}, the indices are rotated starting with the subscript of the matrix. For details, see ref.[9]. Ion activities a_K (instead of concentrations, see ref.[3]) are inserted into the binding reactions by

$$k_{12} = k_{12,1} \cdot a_{\text{Kin}}, \tag{S7a}$$

$$k_{54} = k_{54,1} \cdot a_{\text{Kout}}. \tag{S7b}$$

The rate constants with index "1" are the rate constants for a_K = 1 mM. Voltage sensitivity is introduced by

$$k_{23} = k_{23,0} \exp\left(s_{23} V / V_{23}\right), \tag{S8a}$$

$$k_{32} = k_{32,0} \exp\left(\left(s_{23} - 1\right) V / V_{23}\right), \tag{S8b}$$

and

$$k_{51} = k_{51,0} \exp\left(s_{51} V / V_{51}\right), \tag{S9a}$$

$$k_{15} = k_{15,0} \exp\left(\left(s_{51} - 1\right) V / V_{51}\right). \tag{S9b}$$

s_{ij} are the locations of the Eyring barriers and V_{ij} the characteristic voltages (causing an *e*-fold increase) of the respective state transitions. The rate constants with index "0" are the rate constants at 0 mV. In previous work, the parameters of this model were determined for Kcv_{NTS} under control conditions without blocker[3]. The parameters are shown in Table S1.

Supplementary Table S1. Parameters for the model in Fig. 3A as obtained previously[3] from the analysis of IV curves and gating. The error is given as geometrical error (scatter factor, which gives the standard deviation as a factor). "calc" indicates that k_{21} is not a fit parameter as it can be calculated from micro reversibility at 1 mM K^+ on either side and V = 0 mV: $k_{21} = k_{12,1}\ k_{23,0}\ k_{34}\ k_{45}\ k_{51,0}$ / ($k_{32,0}\ k_{43}\ k_{54,1}\ k_{15,0}$). The rate constants $k_{12,1}$ and k_{21} are labelled by *, because these values are not well determined. Only the ratio $k_{12,1}/k_{21}$ (last row) is reliable.

Parameter	Unit	Geometric mean	Scatter factor
$k_{12,1}$ (a_{Kin})	μs^{-1} mM^{-1}	105*	
k_{21} (*calc*)	μs^{-1}	59768*	
$k_{23,0}$ (V)	μs^{-1}	1214	1.08
$k_{32,0}$ (V)	μs^{-1}	1941	1.38
k_{34}	μs^{-1}	252	1.12
k_{43}	μs^{-1}	685	1.21
k_{45}	μs^{-1}	6824	2.49
$k_{54,1}$ (a_{Kout})	μs^{-1} mM^{-1}	35.4	2.07
$k_{51,0}$ (V)	μs^{-1}	1694	1.04
$k_{15,0}$ (V)	μs^{-1}	132.6	1.05
s_{23}		0.24	1.30
V_{23}	mV	55.9	1.04
s_{15}		0.40	1.11
V_{15}	mV	44.9	1.06
$k_{12,1}/k_{21}$		0.0018	1.14

Ion occupancy measures from 3D RISM (reference interaction site model) calculations

The thermodynamic constants are obtained from the molecule-solvent site pair distribution function $g(\mathbf{r})$ on a grid $\mathbf{r}$ as a result of a solute-solvent interaction model taken from an established force field as in molecular dynamics (MD) simulations. Unlike MD, we quickly obtain from these calculations the equilibrium populations, i.e. the ion occupation probabilities under the many conditions that were used in the experiments. At the price of neglected solute flexibility this analysis offers reliable trends on ion occupations in the SF under noise-free controlled conditions[10].

The equilibrium occupancy of K^+ in the conductive pathway can be calculated by two thermodynamic routes: In a first scenario the channel is exposed to a finite concentration solvent (as in ref.[10]; so-called sol<u>u</u>te-sol<u>v</u>ent (*uv*) calculations) which provides directly a partitioning constant K_c (mass action law) for species i according to

$$K_{c,i} = \frac{c_{\mathrm{ch},i}}{c_{\mathrm{bulk},i}} = \frac{N_{\mathrm{ch},i}}{V_{\mathrm{ch}}\rho_i} = V_{\mathrm{ch}}^{-1}\int_{V_{\mathrm{ch}}} g_i^{uv}(\mathbf{r})\,d\mathbf{r}\,. \tag{S10}$$

with c_{ch} being the concentration within the channel, c_{bulk} the reference pure solvent ion concentration, N_{ch} the number of particles in the channel volume V_{ch}, and ρ the bulk density. If the integration is performed over two-dimensional slices orthogonal to the z coordinate, the integration over z can be done cumulatively starting at a reference point to yield the total partitioning constant.

In a second scenario, we can also start from the perspective of a reaction between an infinitely diluted (single hydrated) ion and the hydrated channel to derive a thermodynamic binding constant K according to

$$K_i = \rho^0 \int_{V_{\mathrm{ch}}} g_i^{uu}(\mathbf{r})\,d\mathbf{r}\,. \tag{S11}$$

from a so-called sol<u>u</u>te-sol<u>u</u>te (*uu*) calculation[11,12] where $\rho^0 = 6.02214\ 10^{-4}$ Å^{-3} corresponds to the formal standard concentration $c^0 = 1$ mol l^{-1}.

The influence of the blocker on the ion distribution in the selectivity filter

3D RISM calculations were performed with standard force field parameters (see main text) for the channel protein as well as with scaled carbonyl charges. TBA parameters are collected in Table S2. The calculations with reduced filter charges were performed by halving the carbonyl atom charges while keeping the total charge of the protein constant. This leads to a partial charge of the carbonyl C atom from original 0.51 to 0.255 and the carbonyl O atom from -0.51 to -0.255 (Table S3). Radial integration was performed based on grid data sliced by applying the HOLE algorithm[13].

Supplementary Table S2. TBA parameters used for the calculations, atom notation following the pdb file.

Atom	q / e	ε / zJ	σ / Å
N1	-0.6048	3.24999852377596	1.181108965
C11	0.1113	3.39966950842353	0.760078358
C12	-0.10215	3.39966950842353	0.760078358
C21	0.1113	3.39966950842353	0.760078358
C22	-0.10215	3.39966950842353	0.760078358
C31	0.1113	3.39966950842353	0.760078358
C32	-0.10215	3.39966950842353	0.760078358
C41	0.1113	3.39966950842353	0.760078358
C42	-0.10215	3.39966950842353	0.760078358
C13	-0.0814	3.39966950842353	0.760078358
C14	-0.09935	3.39966950842353	0.760078358
C23	-0.0814	3.39966950842353	0.760078358
C24	-0.09935	3.39966950842353	0.760078358
C33	-0.0814	3.39966950842353	0.760078358
C34	-0.09935	3.39966950842353	0.760078358
C43	-0.0814	3.39966950842353	0.760078358
C44	-0.09935	3.39966950842353	0.760078358
H443	0.049867	2.64953278774937	0.109078887
H442	0.049867	2.64953278774937	0.109078887
H441	0.049867	2.64953278774937	0.109078887
H432	0.05095	2.64953278774937	0.109078887
H431	0.05095	2.64953278774937	0.109078887
H422	0.06595	2.64953278774937	0.109078887
H421	0.06595	2.64953278774937	0.109078887
H412	0.094825	1.95997717990875	0.109078887
H411	0.094825	1.95997717990875	0.109078887
H343	0.049867	2.64953278774937	0.109078887
H342	0.049867	2.64953278774937	0.109078887
H341	0.049867	2.64953278774937	0.109078887
H332	0.05095	2.64953278774937	0.109078887
H331	0.05095	2.64953278774937	0.109078887
H322	0.06595	2.64953278774937	0.109078887
H321	0.06595	2.64953278774937	0.109078887
H312	0.094825	1.95997717990875	0.109078887

H311	0.094825	1.95997717990875	0.109078887
H243	0.049867	2.64953278774937	0.109078887
H242	0.049867	2.64953278774937	0.109078887
H241	0.049867	2.64953278774937	0.109078887
H232	0.05095	2.64953278774937	0.109078887
H231	0.05095	2.64953278774937	0.109078887
H222	0.06595	2.64953278774937	0.109078887
H221	0.06595	2.64953278774937	0.109078887
H143	0.049867	1.95997717990875	0.109078887
H142	0.049867	1.95997717990875	0.109078887
H141	0.049867	2.64953278774937	0.109078887
H132	0.05095	2.64953278774937	0.109078887
H131	0.05095	2.64953278774937	0.109078887
H122	0.06595	2.64953278774937	0.109078887
H121	0.06595	2.64953278774937	0.109078887
H112	0.094825	2.64953278774937	0.109078887
H111	0.094825	2.64953278774937	0.109078887

Supplementary Table S3. Filter carbonyl atoms with the corresponding index from the KcsA pdb file (2HVK) with original and scaled (to half of the original value) charges. Identical parameters were used for all reduced filter models.

Monomer	Atom	Index	q_{orig} / e_0	$q_{reduced}$ / e_0
Monomer 1	C	805	0.510	0.255
	O	806	-0.510	-0.255
	C	819	0.510	0.255
	O	820	-0.510	-0.255
	C	835	0.510	0.255
	O	836	-0.510	-0.255
	C	842	0.510	0.255
	O	843	-0.510	-0.255
	C	863	0.510	0.255
	O	864	-0.510	-0.255
	C	870	0.510	0.255
	O	871	-0.510	-0.255
Monomer 2	C	2375	0.510	0.255
	O	2376	-0.510	-0.255
	C	2389	0.510	0.255
	O	2390	-0.510	-0.255
	C	2405	0.510	0.255
	O	2406	-0.510	-0.255
	C	2433	0.510	0.255
	O	2434	-0.510	-0.255
	C	2440	0.510	0.255
	O	2441	-0.510	-0.255
Monomer 3	C	3945	0.510	0.255
	O	3946	-0.510	-0.255
	C	3959	0.510	0.255
	O	3960	-0.510	-0.255

	C	3975	0.510	0.255
	O	3976	-0.510	-0.255
	C	4003	0.510	0.255
	O	4004	-0.510	-0.255
	C	4010	0.510	0.255
	O	4011	-0.510	-0.255
Monomer 4	C	5515	0.510	0.255
	O	5516	-0.510	-0.255
	C	5529	0.510	0.255
	O	5530	-0.510	-0.255
	C	5545	0.510	0.255
	O	5546	-0.510	-0.255
	C	5573	0.510	0.255
	O	5574	-0.510	-0.255
	C	5580	0.510	0.255
	O	5581	-0.510	-0.255

Supplementary Table S4. Filter carbonyl atom positions of the reduced model of HEVK.

Atom	*x* /Å	*y* /Å	*z* /Å
C	-3.996	-3.868	2.790
O	-3.816	-2.719	2.414
C	-2.250	-2.845	5.118
O	-1.390	-2.047	5.351
C	-2.995	-1.914	7.989
O	-2.468	-0.974	8.539
C	-2.411	-2.580	10.983
O	-1.404	-2.123	11.527
C	-2.964	-1.920	13.859
O	-2.622	-1.055	14.655
C	-2.727	-4.085	16.273
O	-2.231	-4.387	17.358
C	3.996	3.868	2.790
O	3.816	2.719	2.414
C	2.250	2.845	5.118
O	1.390	2.047	5.351
C	2.995	1.914	7.989
O	2.468	0.974	8.539
C	2.411	2.580	10.983
O	1.404	2.123	11.527
C	2.964	1.920	13.859
O	2.622	1.055	14.655
C	2.727	4.085	16.273
O	2.231	4.387	17.358
C	3.868	-3.996	2.790
O	2.719	-3.816	2.414
C	2.845	-2.250	5.118
O	2.047	-1.390	5.351

C	1.914	-2.995	7.989
O	0.974	-2.468	8.539
C	2.580	-2.411	10.983
O	2.123	-1.404	11.527
C	1.920	-2.964	13.859
O	1.055	-2.622	14.655
C	4.085	-2.727	16.273
O	4.387	-2.231	17.358
C	-3.868	3.996	2.790
O	-2.719	3.816	2.414
C	-2.845	2.250	5.118
O	-2.047	1.390	5.351
C	-1.914	2.995	7.989
O	-0.974	2.468	8.539
C	-2.580	2.411	10.983
O	-2.123	1.404	11.527
C	-1.920	2.964	13.859
O	-1.055	2.622	14.655
C	-4.085	2.727	16.273
O	-4.387	2.231	17.358

Supplementary Table S5. Filter carbonyl atom positions of the reduced model of 1K4C.

Atom	*x* /Å	*y* /Å	*z* /Å
C	-3.987	-3.889	2.791
O	-3.822	-2.767	2.324
C	-2.161	-2.717	5.139
O	-1.279	-1.892	5.374
C	-2.866	-1.830	7.991
O	-2.161	-0.934	8.460
C	-2.315	-2.479	10.980
O	-1.294	-1.952	11.450
C	-2.848	-1.853	13.873
O	-2.364	-0.978	14.570
C	-2.686	-4.030	16.368
O	-2.189	-4.299	17.460
C	3.954	3.860	2.775
O	3.955	2.685	2.440
C	2.135	2.691	5.130
O	1.273	1.857	5.330
C	2.850	1.808	7.980
O	2.276	0.885	8.524
C	2.307	2.460	10.969
O	1.295	2.067	11.514
C	2.849	1.837	13.862
O	2.508	0.985	14.672
C	2.695	4.017	16.355
O	2.205	4.309	17.443

C	3.866	-3.991	2.772
O	2.736	-3.819	2.314
C	2.691	-2.155	5.120
O	1.867	-1.279	5.369
C	1.782	-2.877	7.979
O	0.915	-2.164	8.455
C	2.455	-2.305	10.950
O	1.939	-1.300	11.445
C	1.824	-2.859	13.841
O	0.972	-2.374	14.565
C	3.988	-2.682	16.361
O	4.300	-2.201	17.449
C	-3.891	3.956	2.787
O	-2.726	3.914	2.422
C	-2.717	2.135	5.138
O	-1.881	1.275	5.340
C	-1.828	2.846	7.987
O	-0.912	2.231	8.500
C	-2.474	2.301	10.977
O	-2.066	1.290	11.514
C	-1.844	2.841	13.869
O	-0.990	2.506	14.679
C	-4.019	2.683	16.366
O	-4.315	2.191	17.451

Supplementary Table S6. Filter carbonyl atom positions of the reduced model of 3ZRS.

Atom	*x* /Å	*y* /Å	*z* /Å
C	-3.829	-3.809	2.815
O	-3.565	-2.696	2.344
C	-2.095	-2.705	5.197
O	-1.286	-1.766	5.273
C	-2.835	-1.865	8.127
O	-2.146	-0.930	8.561
C	-2.335	-2.472	11.020
O	-1.371	-1.764	11.327
C	-2.982	-1.802	13.876
O	-2.387	-0.917	14.488
C	-2.777	-4.101	16.458
O	-2.110	-4.498	17.419
C	3.797	-3.837	2.813
O	2.684	-3.574	2.344
C	2.697	-2.101	5.195
O	1.758	-1.291	5.272
C	1.863	-2.836	8.127
O	0.928	-2.147	8.562
C	2.474	-2.334	11.019
O	1.767	-1.369	11.326

C	1.809	-2.977	13.877
O	0.925	-2.381	14.489
C	4.112	-2.769	16.454
O	4.511	-2.101	17.414
C	3.826	3.789	2.802
O	3.562	2.676	2.335
C	2.093	2.692	5.188
O	1.284	1.753	5.267
C	2.834	1.861	8.120
O	2.145	0.926	8.557
C	2.336	2.476	11.012
O	1.372	1.769	11.321
C	2.984	1.814	13.870
O	2.389	0.930	14.484
C	2.780	4.120	16.445
O	2.114	4.520	17.405
C	-3.801	3.818	2.804
O	-2.688	3.553	2.335
C	-2.699	2.088	5.190
O	-1.760	1.279	5.269
C	-1.863	2.832	8.120
O	-0.928	2.144	8.556
C	-2.473	2.337	11.014
O	-1.766	1.374	11.323
C	-1.807	2.989	13.869
O	-0.922	2.395	14.483
C	-4.109	2.788	16.448
O	-4.507	2.123	17.410

Supplementary Table S7. Filter carbonyl atom positions of the reduced model of $Kcv_{PBCV\text{-}1}$.

Atom	*x* /Å	*y* /Å	*z* /Å
C	-4.147	-5.098	3.367
O	-3.991	-4.015	2.807
C	-2.537	-3.426	5.316
O	-1.843	-2.395	5.331
C	-2.839	-1.961	7.943
O	-2.186	-0.996	8.310
C	-2.788	-1.980	11.000
O	-2.121	-1.097	11.521
C	-3.271	-1.583	13.724
O	-2.567	-0.637	13.920
C	-2.874	-2.961	16.711
O	-2.339	-2.845	17.795
C	5.049	-4.526	3.335
O	3.982	-4.262	2.785
C	3.601	-2.695	5.267
O	2.653	-1.890	5.279

C	2.156	-2.770	7.921
O	1.276	-2.005	8.286
C	2.232	-2.650	10.975
O	1.438	-1.876	11.492
C	1.830	-3.022	13.716
O	0.971	-2.212	13.907
C	3.294	-2.712	16.672
O	3.258	-2.142	17.744
C	4.190	5.329	3.199
O	3.979	4.263	2.627
C	2.514	3.718	5.144
O	1.774	2.719	5.154
C	2.762	2.220	7.758
O	2.068	1.282	8.122
C	2.726	2.216	10.815
O	2.023	1.361	11.334
C	3.205	1.777	13.534
O	2.458	0.862	13.726
C	2.886	3.148	16.534
O	2.351	3.049	17.620
C	-4.964	4.139	3.230
O	-3.887	3.886	2.696
C	-3.504	2.363	5.205
O	-2.542	1.576	5.238
C	-2.090	2.505	7.872
O	-1.199	1.762	8.258
C	-2.196	2.430	10.926
O	-1.393	1.679	11.464
C	-1.831	2.852	13.666
O	-0.960	2.060	13.878
C	-3.321	2.559	16.611
O	-3.286	2.007	17.692

Supplementary references

1. Schroeder, I. How to resolve microsecond current fluctuations in single ion channels: The power of beta distributions. *Channels* **9**, 262–280 (2015).
2. Rauh, O. *et al.* Extended beta distributions open the access to fast gating in bilayer experiments - assigning the voltage-dependent gating to the selectivity filter. *FEBS Lett.* **591**, 3850–3860 (2017).
3. Rauh, O., Hansen, U.-P., Scheub, D. D., Thiel, G. & Schroeder, I. Site-specific ion occupation in the selectivity filter causes voltage-dependent gating in a viral K^+ channel. *Sci. Rep.* **8**, 10406 (2018).
4. Schroeder, I. & Hansen, U.-P. Using a five-state model for fitting amplitude histograms from MaxiK channels: beta-distributions reveal more than expected. *Eur. Biophys. J.* **38**, 1101–1114 (2009).
5. Hille, B. Ion Channel Excitable Membranes. *Sunderland Massachusetts USA* 1–37 (2001). doi:10.1007/3-540-29623-9_5640
6. Schroeder, I. & Hansen, U.-P. Saturation and microsecond gating of current indicate depletion-induced instability of the MaxiK selectivity filter. *J. Gen. Physiol.* **130**, 83–97 (2007).
7. Albertsen, A. & Hansen, U.-P. Estimation of kinetic rate constants from multi-channel recordings by a direct fit of the time series. *Biophys. J.* **67**, 1393–1403 (1994).
8. Läuger, P. Internal motions in proteins and gating kinetics of ionic channels. *Biophys. J.* **53**, 877–884 (1988).
9. Hansen, U.-P., Rauh, O. & Schroeder, I. A simple recipe for setting up the flux equations of cyclic and linear reaction schemes of ion transport with a high number of states: the arrow scheme. *Channels* **10**, 1–20 (2016).
10. Kast, S. M., Kloss, T., Tayefeh, S. & Thiel, G. A minimalist model for ion partitioning and competition in a K^+ channel selectivity filter. *J. Gen. Physiol.* **138**, 371–373 (2011).
11. Kloss, T. & Kast, S. M. Treatment of charged solutes in three-dimensional integral equation theory. *J. Chem. Phys.* **128**, 134505 (2008).
12. Mrugalla, F. & Kast, S. M. Designing molecular complexes using free-energy derivatives from liquid-state integral equation theory. *J. Phys. Condens. Matter* **28**, 344004 (2016).
13. Smart, O. S., Neduvelil, J. G., Wang, X., Wallace, B. A. & Sansom, M. S. P. HOLE: A program for the analysis of the pore dimensions of ion channel structural models. *J. Mol. Graph.* **14**, 354–360 (1996).
